# Physical geography, isolation by distance and environmental variables shape genomic variation of wild barley (*Hordeum vulgare* L. ssp. *spontaneum*) in the Southern Levant

**DOI:** 10.1038/s41437-021-00494-x

**Published:** 2022-01-11

**Authors:** Che-Wei Chang, Eyal Fridman, Martin Mascher, Axel Himmelbach, Karl Schmid

**Affiliations:** 1 University of Hohenheim, Stuttgart, Germany; 2 Plant Sciences Institute, Agricultural Research Organization (ARO), The Volcani Center, Rishon LeZion, Israel; 3 Leibniz Institute of Plant Genetics and Crop Plant Research (IPK), Seeland OT Gatersleben, Germany

**Keywords:** Genetic variation, Agriculture, Evolutionary ecology, Evolutionary genetics, Plant evolution

## Abstract

Determining the extent of genetic variation that reflects local adaptation in crop-wild relatives is of interest for the purpose of identifying useful genetic diversity for plant breeding. We investigated the association of genomic variation with geographical and environmental factors in wild barley (*Hordeum vulgare L. ssp. spontaneum*) populations of the Southern Levant using genotyping by sequencing (GBS) of 244 accessions in the Barley 1K+ collection. The inference of population structure resulted in four genetic clusters that corresponded to eco-geographical habitats and a significant association between lower gene flow rates and geographical barriers, e.g. the Judaean Mountains and the Sea of Galilee. Redundancy analysis (RDA) revealed that spatial autocorrelation explained 45% and environmental variables explained 15% of total genomic variation. Only 4.5% of genomic variation was solely attributed to environmental variation if the component confounded with spatial autocorrelation was excluded. A synthetic environmental variable combining latitude, solar radiation, and accumulated precipitation explained the highest proportion of genomic variation (3.9%). When conditioned on population structure, soil water capacity was the most important environmental variable explaining 1.18% of genomic variation. Genome scans with outlier analysis and genome-environment association studies were conducted to identify adaptation signatures. RDA and outlier methods jointly detected selection signatures in the pericentromeric regions, which have reduced recombination, of the chromosomes 3H, 4H, and 5H. However, selection signatures mostly disappeared after correction for population structure. In conclusion, adaptation to the highly diverse environments of the Southern Levant over short geographical ranges had a limited effect on the genomic diversity of wild barley. This highlighted the importance of nonselective forces in genetic differentiation.

## Introduction

Local adaptation is an essential survival strategy for plants in stressful environments because they are sessile. Natural selection in heterogeneous environments leads to increased fitness of local genotypes. Gene flow can, however, offset genetic differentiation resulting from local adaptation and reduce fitness (Kawecki and Ebert 2004). In addition, genetic drift and demographic history contribute to genetic differentiation and confound adaptive variation with neutral variation (Kawecki and Ebert 2004; Günther and Coop 2013; López-Goldar and Agrawal 2021). Consequently, the combination of selective and nonselective forces simultaneously shapes genetic variation and leads to geographical patterns of population divergence and allele frequency distribution. Determining how different population genetic processes affect the geographical distribution of genetic variation is a key component in the study of plant adaptation. Investigating the role of adaptive and non-adaptive processes in genomic variation is of particular interest for wild relatives of crop plants, as this may allow the discovery of useful genetic variation for plant breeding (Turner-Hissong et al. 2020).

Wild barley (*Hordeum vulgare L. ssp. spontaneum*) is a highly suitable model species for studying the local adaptation of crop-wild relatives, as it occurs over a wide geographical range in the Fertile Crescent and Central Asia (Harlan and Zohary 1966). Within this range, genotypes originating in Central Asia are genetically clustered with those from the eastern Fertile Crescent (Jakob et al. 2014; Russell et al. 2016; Pankin et al. 2018). There is a tendency of increasing genetic diversity from the east toward the west (Jakob et al. 2014). Wild barley in the western Fertile Crescent, i.e., the Levant, has the highest genetic diversity of the Fertile Crescent (Jakob et al. 2014; Russell et al. 2016; Pankin et al. 2018). It occupies heterogeneous environments, including Mediterranean and desert climates, within a short geographical distance (Hübner et al. 2009, Nevo et al. 1979, Volis et al. 2001). Wild barley populations from the Southern Levant show a strong correlation between genetic and environmental distances (Hübner et al., 2009). Population structure reflects eco-geographical habitats (Hübner et al. 2009, 2012) and distinguishes between northern and southern genetic clusters correlated with latitude and precipitation gradients (Jakob et al. 2014; Russell et al. 2016). Common garden experiments in previous studies revealed that eco-geography was correlated with morphological traits (Hübner et al. 2013), phenotypic plasticity (Galkin et al. 2018), and rhizosphere microbiota (Terrazas et al. 2020). Moreover, transplantation experiments showed a correlation between the geographical origin of wild barley ecotypes and fitness in different environments, suggesting local adaptation (Volis et al. 2002a, b, 2011). In addition to a broad geographical scale, environmental differences on a fine geographical scale also contribute to genetic diversification in wild barley (Nevo et al. 2005; Bedada et al. 2014; Wang et al. 2018). Overall, these results suggest a strong relationship between environmental differences, genetic divergence and phenotypic diversity of wild barley populations. This supports the hypothesis of local adaptation of wild barley in the Southern Levant. However, the relative contributions of environmental and nonselective forces to genetic variation and the genetic architecture of adaptive traits are still mostly unclear due to the lack of appropriate statistical approaches, fine-scale environmental data, and sufficient genome-wide markers.

Wild barley is a valuable genetic resource for barley breeding because domestication and modern breeding have greatly reduced the genetic diversity of cultivated barley (*H. vulgare L. ssp. vulgare*; Caldwell et al. 2006, Kilian et al. 2006). Since wild barley has no reproductive barrier to cultivated barley (Nevo et al. 1979), the genetic diversity of cultivated barley can be enhanced by introducing alleles from wild populations (Dawson et al. 2015). Numerous studies have shown evidence for local adaptation of wild barley to different environments (Nevo et al. 1979, Hübner et al. 2013, Galkin et al. 2018, Volis et al. 2002a, b, Bedada et al. 2014; Volis et al. 2004, 2011; Wang et al. 2018). Wild barley is, therefore, expected to possess considerable genetic variation that contributes to adaptation to various abiotic stresses (Dawson et al. 2015). Correspondingly, wild barley has been used as a source of novel alleles to improve stress tolerance in barley breeding (Baum et al. 2003; Pham et al. 2019). However, widespread use of wild barley has been limited due to its large genome size (~5.3 Gb) and undesirable traits (Schmid et al. 2018). To facilitate the utilization of favorable alleles in wild barley, it is important to take advantage of novel genomic technologies and eco-geographical information. Insight into the association between genetic variation and environments provides information that can help to guide the identification of valuable germplasms and the selection of core accessions for generating introgression lines and carrying out further genome-trait association studies (Bohra et al. 2021).

Genotyping by sequencing (GBS; Elshire et al. 2011; Poland et al. 2012) and a high-quality barley genome assembly (Mascher et al. 2017; Jayakodi et al. 2020) permit the exploration of genomic variation under environmental selection and the search for useful genetic variation in wild barley. In this study, we investigated genetic variation with high-density genome-wide markers that had not been used in earlier studies of wild barley in the Levant. We aimed to (1) describe the population structure of wild barley from the Southern Levant and place it in the context of a worldwide sample (Milner et al. 2019), (2) examine geographical patterns of gene flow in the Southern Levant, (3) characterize the relative contributions of environmental gradients and space to genomic variation and population structure, and (4) identify putative adaptive loci. Overall, our results indicated that geography and spatial autocorrelation were more important than selection for local adaptation in shaping genomic variation in wild barley in the Southern Levant. However, diverse environments, particularly water availability, show significant associations with genetic differentiation.

## Materials and methods

### Plant material and genotyping by sequencing

**Fig. 1:**
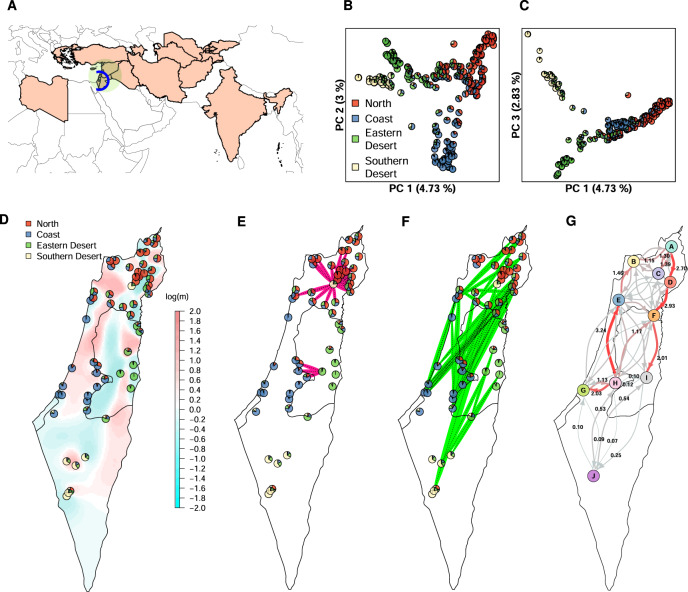
Spatial genetic structure of 244 B1K+ accessions and results of gene flow analysis. **A** A geographical map of accession origins. Countries of origin of IPK accessions are colored in light red. The green close circle represents the Levant region. The blue open circle indicates the origin of B1K+ collection. **B** PCA plot of the first and second PC axes. **C** PCA plot of the first and third PC axes. Pie charts in PCA plots represent ancestry coefficients of individuals estimated by *ALStructure* with *K* *=* 4. **D** Distribution of genetic clusters and effective migration surface. Pie charts give the average ancestry coefficients of individuals in collection sites. Color gradient represents gene flow rates estimated by *EEMS*. **E** Population pairs with *unPC* scores higher than the top 2.5% threshold which indicate a significantly low genetic similarity over a short geographical distance. **F** Population pairs with *unPC* scores lower than the bottom 2.5% threshold which indicate significantly high genetic similarity over a large geographical distance. **G** Gene flow rates inferred by the coalescent-based inference method, representing the probabilities per unit of time that individuals in a region *i* are descended from a region *j* (Lundgren and Ralph 2019). The thickness of arrows and the depth of red color is proportional to gene flow rates. The full results are given in Table S4.

We genotyped 244 wild barley accessions collected in the Southern Levant region (Fig. 1A). These accessions, hereafter referred to as B1K+ accessions, included 191 accessions from Barley 1K (B1K) collection (Hübner et al. 2009) and 53 accessions from an unpublished collection referred to as HOH, collected in 2005, 2009, and 2011 by K.S. (Fig. S1; File S1). The GBS library was constructed using genomic DNA digested with the restriction enzyme *PstI* and *MspI* as in Milner et al. (2019). In addition, published GBS data of 1121 wild barley accessions from the IPK genebank (Milner et al. 2019) were included (Fig. 1A). Because the IPK genebank contains accessions from Israel, we specified the source between IPK and B1K+ accessions to avoid confusion. Identification of single-nucleotide polymorphism (SNP) was performed as Milner et al. (2019). The detailed workflow of genotypic data filtration is described in Supplementary Text and summarized in Fig. S2.

### Environmental data

To investigate the relationship between genetic variation and environmental gradients, we used environmental data including (1) climate data from the *WorldClim2* database (Fick and Hijmans 2017) with a resolution of 30 arcseconds [~1 km], (2) soil data from the *SoilGrids* database (Hengl et al. 2017) with a resolution of 250m, (3) topographic variables based on elevation data from the *SRTM* database (https://srtm.csi.cgiar.org/) with a resolution of 90m, and (4) geographical coordinates of collection points (Supplementary Text; File S1). To mitigate the problem of collinearity for redundancy analysis (RDA; Legendre and Legendre 2012), highly correlated environmental variables were grouped by hierarchical clustering using a customized clustering index (Supplementary Text; Fig. S3). We then selected the eigenvector of the first principal axis for each collinear group as a synthetic variable to represent highly correlated environmental variables. Next, all environmental variables, including the synthetic variables, were selected based on variance inflation factors (VIFs) until all VIFs were less than 5. Details of this procedure are given in Supplementary Text. Finally, 12 environmental variables, including 7 synthetic and 5 nonsynthetic variables (Tables S1 and S2), were selected for environmental association analyses.

### Inference of population structure

The number of ancestors and ancestry coefficients were estimated using the model-based method *ALStructure* (Cabreros and Storey 2019). *ALStructure* uses a likelihood-free algorithm to derive estimates from minimal model assumptions. It is generally superior to existing likelihood-based methods in terms of accuracy and computational speed. The method does not assume Hardy-Weinberg equilibrium within populations, but defines the number of ancestral populations (*K*) as the rank of a matrix consisting of individual-specific allele frequencies (Leek 2011). Optimal *K* was calculated using the *estimate_d* function of the R package *alstructure*(Cabreros and Storey 2019), and ancestry coefficients were estimated using the *alstructure* function. A range of *K* values, from 2 to 8, was also used to examine the stratification of population structure. In addition to *ALStructure*, principal component analysis (PCA) and neighbor-joining (NJ) were also performed. Missing genotypic values (~3% of the dataset) were replaced by the average number of alternative alleles at each SNP locus before performing PCA.

To analyze genetic differentiation, we calculated *F*_*ST*_ and Nei genetic distance between genetic clusters defined by *ALStructure*. Accessions were assigned to genetic clusters according to the highest ancestry coefficient calculated by *ALStructure* with the optimal *K* value. *F*_*ST*_ values were calculated as ratio of average values (Bhatia et al. 2013) and Nei’s genetic distances were calculated using the function *stamppNeisD* of the R package *StAMPP* (Pembleton et al. 2013).

### Analysis of gene flow

To identify gene flow barriers that may explain observed population structure, an analysis of estimated effective migration surfaces (*EEMS*; Petkova et al. 2016) was done. First, B1K+ accessions were clustered into 58 demes that correspond to the location of collection sites. *EEMS* was then conducted in three independent runs of Markov chain Monte Carlo (MCMC), and the results of the three runs were averaged. Each MCMC chain encompassed 10 million burn-in iterations and 10 million post-burn-in iterations thinned by an interval of 5000 iterations. Outputs of *EEMS* were processed using the R package *rEEMSplots*. To examine whether geographical barriers contribute to genetic isolation, we separated map pixels into barrier and non-barrier pixels according to geographical elevation (details in Supplementary Text; Fig. S4). We then conducted a Wilcoxon test to examine the hypothesis that geographical barriers are significantly associated with lower gene flow rates.

To account for non-independence between observations, we used *ResistanceGA* (Peterman 2018) to assess the support for isolation by geographical barriers. In short, *ResistanceGA* optimizes resistance surfaces based on genetic distances and transformed landscape features with a genetic algorithm. Pairwise genetic distances between 58 demes were calculated as *D*_ps_ = 1 − *p*, where *p* is the proportion of shared alleles. In the analysis of *ResistanceGA*, we converted elevation and slope to continuous resistance surfaces, respectively, with inverse ricker and inverse-reverse monomolecular transformation. In addition, surface water data obtained from Global-Surface Water (https://global-surface-water.appspot.com/download; Pekel et al. 2016) were used as the categorical resistance surface for *ResistanceGA*. To assess the model fit of different landscape feature combinations, we carried out two bootstrap analyses that separately used *R*^2^ and Akaike information criterion (AIC) as the model ranking standard with 1,000 iterations. Default parameters were used for the *ResistanceGA* framework.

As a complementary method to *EEMS*, unbundled principal components (unPC; House and Hahn 2018) were employed to reveal potential long-distance migration. *unPC* scores, a ratio of PCA-based genetic distance on population level to geographical distance between demes, were computed with the R package *unPC*. Original *unPC* scores were transformed by Box–Cox transformation into an approximate Gaussian distribution. Subsequently, an outlier test based on Student’s t-distribution with a two-tailed significance level of 0.05 was performed to identify extreme population pairs. In this test, we assumed dependence between genetic and geographical distances. A null hypothesis is that samples from a pair of collection sites display an isolation-by-distance pattern. In other words, outliers identified with this test are considered to result from the violation of isolation-by-distance, which could constitute either long-distance migration or isolation due to unknown factors.

To infer asymmetric gene flows, we utilized the coalescent-based inference (CBI; Lundgren and Ralph 2019). We manually grouped accessions into ten geographical regions (Fig. S5) such that each region covered roughly equal geographical areas as suggested by Lundgren and Ralph (2019). We likewise considered the gene flow pattern inferred by *EEMS*. The sample sizes in each region ranged from 10 to 41 with an average of 24.4. Next, we created an adjacency matrix to allow gene flow between adjacent regions (Fig. S5). For CBI input, pairwise genetic distances were computed as the average number of different alleles across SNPs. CBI was performed using the R package *gene.flow.inference* (Lundgren and Ralph 2019) with 2 million pre-burn-in iterations, 60 million burn-in iterations, and 100 million post-burn-in iterations followed by a thinning process for every 5000 iterations to rule out serial correlations. Medians of gene flow rates and coalescence rates were computed from posterior distributions and 95% credible intervals were calculated with the highest density interval method by using the R package *bayestestR* (Makowski et al. 2019).

### Partitioning genomic variation

To partition genomic variation into components explained by different factors, we conducted RDA, a multivariate method for studying a linear relationship between two or more matrices (Legendre and Legendre 2012). Specifically, we used simple RDA and RDA conditioned on covariates, i.e., partial RDA, to estimate the proportion of SNP variation explained by environmental variables, spatial autocorrelation, and population structure. RDA was performed with the *rda* function of the R package *vegan*(Oksanen et al. 2019). For all RDA models in this study, we carried out 5000 permutations to test the significance of explanatory variables with the R function *anova.cca*.

To model the effect of spatial autocorrelation on SNP variation, distance-based Moran’s eigenvector maps (dbMEMs) were used in RDA (Legendre and Legendre 2012; Dray et al. 2006). First, a network of 58 collection sites was built with the Gabriel graph, and a spatial weighting matrix of inverse geographical distances (km^−1^) was constructed in line with the method of Forester et al. (2018). Next, the spatial weighting matrix was decomposed to generate dbMEMs. Subsequently, forward selection was performed to identify dbMEMs that associate significantly with spatial genetic structure by using *forward.sel* function (Dray et al. 2019). The selected dbMEMs with positive and negative eigenvalues, corresponding to broad-scale and fine-scale spatial structures, were both used in RDA to capture comprehensive spatial autocorrelation. In addition, to partition observed population structure, ancestry coefficients estimated by *ALStructure* with the optimal *K* values were used in the RDA on SNPs as covariates.

Since genetic clusters were largely congruent with eco-geographical habitats, we were interested in the degree of population structure that could be attributed to environmental and spatial factors. By fitting RDA models on ancestry coefficients instead of SNPs, we excluded recent genetic variation within populations to better quantify the relative contributions of environments and spatial autocorrelation to population structure. To carry out this analysis, SNPs were replaced by ancestry coefficients inferred by *ALStructure* with the optimal *K* as the new response variables in RDA models.

To evaluate the effects of individual environmental variables on SNP variation, we sequentially fitted one environmental variable at a time as the explanatory variable and treated ancestry coefficients as covariates in RDA models. Considering the correlation between environmental variables (Fig. S3C and D), we conducted additional permutation tests for marginal effects of environmental variables in a model including all environmental variables by setting the parameter *by = ’margin’* for *anova.cca*. This method tested the significance of each environmental variable while removing the confounding effect with the other environmental variables.

### Linkage disequilibrium

Linkage disequilibrium (LD) was evaluated as the pairwise *r*^2^ of SNPs by using the *snpgdsLDMat* of the R package *SNPRelate* (Zheng et al. 2012) with a window size of 250 markers. To evaluate the genome coverage of markers, genome-wide LD decline against physical distance was fitted by using local polynomial regression and the formula of Hill and Weir (1988). Local polynomial regression was carried out by using the R function *loess* with a smoothing parameter of 0.005.

### Identification of selection signatures

As a genome-environment association (GEA) method, RDA has high detection power and a low false-positive rate in identifying adaptation signatures (Forester et al. 2018, 2016; Capblancq et al. 2018). We therefore performed genome scans with simple and partial RDA. Simple RDA was done by treating 27,147 SNPs as response variables and twelve environmental variables as explanatory variables. To control for false positives due to population structure, partial RDA was performed by using ancestry coefficients estimated with the optimal *K* as covariates. A statistical framework proposed by Capblancq et al. (2018) was used for statistical tests and controlling for false discovery rates (FDR). Briefly, the loadings of SNPs in the first four RDA axes, selected according to the proportion of explained variation (Fig. S6), were converted into Mahalanobis distances that approximated to a chi-squared distribution with four degrees of freedom. Next, p-values and q-values were computed accordingly, and SNPs with FDR < 0.05 were considered as candidate adaptive SNPs. The statistical test was conducted using the R function *rdadapt* (Capblancq et al. 2018).

Besides RDA, the latent factor mixed model (LFMM; Caye et al. 2019), which is an univariate GEA method, was performed by using the R package *lfmm* (Caye et al. 2019) with parameter *K* *=* 4 to correct population structure and q-values were subsequently computed. SNPs with FDR < 0.05 were considered to be candidate adaptive SNPs.

As a complement to GEA methods, outlier SNPs with an extreme divergence between genetic clusters were detected by the *X*^*T*^*X* statistics (Günther and Coop 2013). We assigned accessions to genetic clusters according to the highest ancestry coefficient estimated by *ALStructure* with the optimal *K* and calculated *X*^*T*^*X* by using *BAYPASS* ver2.1 (Gautier 2015). *BAYPASS* was run by setting 25 short pilot runs, 100,000 burn-in iterations and 100,000 post-burn-in iterations with a thinning interval of 40 iterations. A significance threshold of *X*^*T*^*X* was determined by the 99.5% quantile of pseudo-observed *X*^*T*^*X* (Gautier 2015) calculated from neutral markers simulated by *simulate.baypass* (Gautier 2015).

### Gene ontology enrichment

To investigate biological functions related to putatively adaptive loci, we conducted gene ontology (GO) enrichment analysis with gene annotations of the barley ’Morex v2’ genome (Mascher 2019). Over-representation of GO terms for genes within 500 bp adjacent intervals of candidate SNPs was tested by Fisher’s exact test with 10,000 runs using *SNP2GO* (Szkiba et al. 2014). GO terms with an FDR < 0.05 were regarded as significantly enriched. Annotations of genes within 500 bp upstream and downstream of the candidate SNPs were also reported.

## Results

### Summary of genotyping data

SNP calling and preliminary filtration resulted in 101,711 SNPs for 1365 accessions, including 1121 IPK accessions and 244 B1K+ accessions. Depending on the analytical requirements, we selected different subsets from 101,711 SNPs as follows. For the joint population structure analysis of IPK and B1K+ accessions, we selected 4,793 SNPs with minor allele frequency (MAF) ≥ 0.05 among 72 IPK accessions originating from 13 countries (Russell et al. 2016). This joint dataset had a missing proportion of 0.043 and was LD-pruned with PLINK using a *r*^2^ threshold of 0.1. For analyses of B1K+ accessions, we selected 58,616 SNPs with an overall missing proportion of 0.029 and maximal individual missing proportion of 0.059. Further filtration resulted in 19,601 SNPs (LD-pruned; MAF ≥ 0.01) and 27,147 SNPs (unpruned; MAF ≥ 0.05; Details in Supplementary Text; Fig. S2)

### Population structure and spatial genetic pattern

**Fig. 2:**
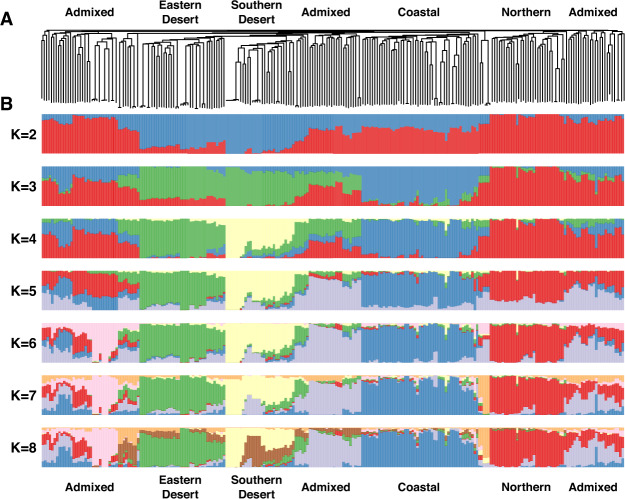
Inference of population structure. **A** Unrooted neighbor-joining (NJ) tree and **B** ancestry coefficients of 244 B1K+ accessions estimated by using *ALStructure* with *K* = 2–8. Accessions are sorted according to the NJ tree. With *K* = 4, red, blue, green, and yellow bars correspond to the Northern, Coastal, Eastern Desert, and Southern Desert genetic clusters, respectively, as in Fig. 1.

The inference of population structure among B1K+ accessions with *ALStructure* (Cabreros and Storey 2019) identified four clusters (Fig. 1B, C) corresponding to the Mediterranean northern region, semi-arid coastal region, Judaean Desert, and Negev Desert (Fig. 1D). Hereafter, we named the four B1K+ clusters as North, Coast, Eastern Desert, and Southern Desert. With *K* *=* 4, 174 of 244 (71.3%) accessions had a highest ancestry coefficient of less than 0.9. The first three principal components (PCs) represented the clusters corresponding to the *ALStructure* results (Fig. 1B, C). On the first PC axis, the northern cluster was separated from two desert clusters, and on the second PC axis, the coastal cluster was separated from the others. On the third PC axis, the southern desert cluster was separated from the eastern desert cluster. The three PC axes explained 4.73%, 3%, and 2.83% of the variation, respectively. A hierarchical population structure was evident in the NJ tree (Fig. 2A) and in the *ALStructure* analysis with *K* = 2–8 (Fig. 2B). To evaluate the importance of marker density and additional samples for population structure analysis, we performed PCA and *ALStructure* by either including or removing the HOH accessions with random selection of 100 and 5000 SNPs, in addition to the original dataset. The dataset with 100 SNPs did not allow to identify genetic clusters while datasets with 5,000 SNPs separated into four genetic clusters by using the first four PCs even without the HOH accessions. However, *ALStructure* could only identify three ancestral populations (*K* = 3) if the HOH accessions were excluded (File S2).

To quantify the extent of genetic differentiation among the four clusters, we computed pairwise *F*_*ST*_ and Nei’s genetic distances. The Southern Desert cluster was most strongly isolated from the other three clusters (Table 1). With respect to the genomic pattern of differentiation, *F*_ST_ values were highest in the pericentromeric regions of the chromosomes 2H, 3H, 4H, 5H, and 6H. The Southern Desert cluster differentiated from the other three clusters in most of the genome except the pericentromeric regions of the chromosomes 3H and 4H (Fig. S7).

**Table 1:** *F*_ST_ and Nei’s genetic distances between four genetic clusters.

		*F*_ST_			
		North	Coast	Eastern desert	Southern desert
	North	–	0.1124	0.1149	0.2593
Nei’s distance	Coast	0.0209	–	0.1321	0.2533
	Eastern Desert	0.0216	0.0248	–	0.2125
	Southern Desert	0.0508	0.0482	0.0389	–

**Fig. 3:**
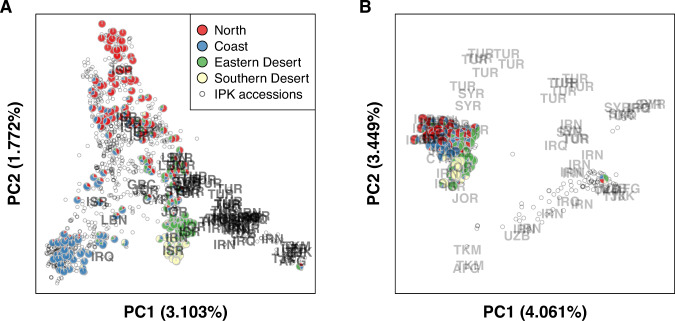
PCA plots of 244 B1K+ accessions and 1121 accessions from the IPK genebank. **A** PCA performed with all of the available accessions. **B** PCA performed with 72 geographically diverse accessions and projection of the remaining accessions to PC spaces. Pie charts represent ancestry coefficients of 244 B1K+ accessions estimated by using *ALStructure* with *K* = 4. Gray open dots represent IPK accessions.

A joint PCA of B1K+ and IPK accessions was consistent with major clusters identified in B1K+ and showed that B1K+ accessions overlapped with a large proportion of the IPK collection (Fig. 3A). To visualize the genetic relationship between B1K+ accessions and IPK accessions of different origins, we selected 72 geographically distinct accessions used in a previous study (Russell et al. 2016). On the first PC axis, most of the 72 geographically diverse accessions collected from western and central Asian countries were separated from B1K+ accessions but clustered more closely to the two desert clusters than to the northern and coastal clusters (Fig. 3A). Because an unbalanced sample size of accessions from Israel (616 out of 1365 accessions) might bias the PC axes, we performed another joint PCA by projecting 1293 accessions onto PC spaces of 72 geographically distinct accessions. The PC projection was done by calculating inner products between genotypic values of 1293 accessions and eigenvectors obtained from the PCA of 72 geographically distinct accessions. This approach could avoid the misinterpretation of sample origin and migration based on PC (McVean 2009). The PC projection showed that accessions typically clustered by geographical origin, as reported in previous studies (Russell et al. 2016; Milner et al. 2019), and B1K+ accessions were concentrated in a small area of PC space (Fig. 3B).

### Geographical pattern of gene flow

To identify barriers limiting gene flow within the Levant region, we performed an *EEMS* (Petkova et al. 2016) analysis that revealed uneven gene flow across the landscape. The area of low gene flow rates corresponded closely to geographical barriers, including the Sea of Galilee, the Jordan Valley, and the Judea and Samaria mountain ridges (Fig. 1D). A Wilcoxon test supported the association between geographical barriers and lower gene flow rates (*p* < 2.2 × 10^−22^; Fig. S8A). In addition, *EEMS* analysis showed that genetic dissimilarity between demes did not have a simple linear relationship with geographical distances (Fig. S8B), indicating that isolation-by-distance was not sufficient to explain genetic differentiation. This result was supported by a *ResistanceGA* analysis indicating that landscape features explained genetic distances better than geographical distances. A composite resistance surface consisting of elevation and slope was suggested as the best predictor according to *R*^2^ in all bootstrap iterations ($\bar{{R}^{2}}=0.51$; Table S3.1), whereas the model of geographical distances had $\bar{{R}^{2}}=0.21$. Model selection with AIC suggested a model with surface water as resistance surface as best model in 96.8% of bootstraps with $\bar{{R}^{2}}=0.31$ (Table S3.2).

The *EEMS* analysis also showed that effective genetic diversity, which is the expected genetic dissimilarity of two individuals sampled from a site (Petkova et al. 2016), decreased from north to south (Fig. S8C), suggesting higher genetic diversity in the north than the south. Furthermore, we performed *unPC* (House and Hahn 2018), the ratio of PC-based genetic distances to geographical distances, which is more sensitive to long-distance migration than *EEMS*. The population pairs with high *unPC* score supported regions of low gene flow identified by *EEMS* (Fig. 1E). This was particularly true for the majority of significant population pairs with located in the region around the Sea of Galilee in northern Israel (Fig. 1E). In addition, the population pairs with low *unPC* scores suggested potentially long-distance migration events in the north-south direction (Fig. 1F).

To evaluate asymmetric gene flows, we used CBI (Lundgren and Ralph 2019), which suggested unequal gene flows in a North-South direction (Fig. 1G; Table S4). There was a trend for gene flow from South (region *H*) to North (region *B*) in the western region (region *H* → *E* → *B*; Fig. 1G) and an opposite trend from North (region *A*) to South (region *I*) in the eastern region (region *A* → *D* → *F* → *I*; Fig. 1G). The strongest gene flow (3.24 with the 95% credible interval of 0.51–6.32; Table S4) was observed from populations close to Jerusalem (region *H*) to the surrounding areas of Mount Carmel (region *E*). However, the gene flow in the opposite direction (region *E* → *H*) was much weaker (0.77 with the 95% credible interval of 0-2.32; Table S4). Low gene flow rates of connections across geographical barriers, such as *C* ⇌ *D* and *H* ⇌ *I*, correlated with the results of *EEMS* (Fig. 1D) and *unPC* (Fig. 1E and F). Furthermore, low gene flow rates between the Negev desert (region *J*) and its adjacent regions indicated the isolation of Southern Desert accessions, consistent with the high genetic differentiation suggested by the *F*_*ST*_ values (Table 1).

### Genetic variation explained by environment and space

#### SNP variation partitioning with redundancy analysis (RDA)

**Fig. 4:**
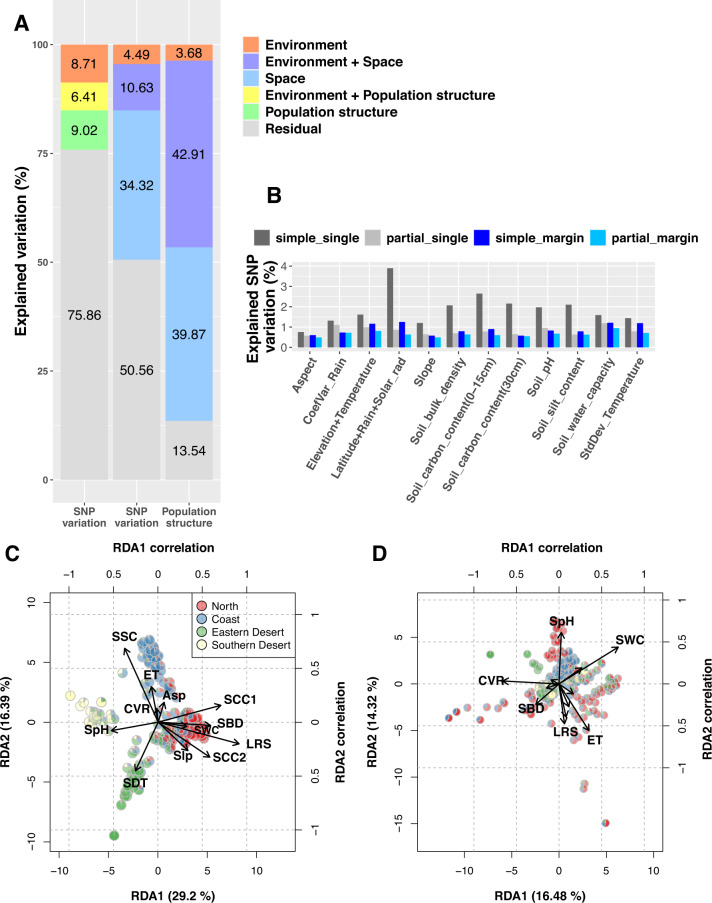
Results of variation partitioning and RDA biplots. **A** Variation partitioning of SNP variation and population structure. Left and middle columns: explained SNP variation estimated by RDA models using population structure and spatial autocorrelation as covariates, respectively. Right column: population structure explained by environment and space. Environment, space and population structure are represented by twelve environmental variables, dbMEMs, and ancestry coefficients (*K* = 4) in RDA models. **B** Percentage of SNP variation explained by environmental variables. The *simple_single* and *partial_single* show individual effects estimated based on RDA models fitting one environmental variable at a time. The *simple_margin* and *partial_margin* show marginal effects estimated based on RDA models fitting all environmental variables. The *partial_single* and *partial_margin* are estimated based on partial RDA conditioned on population structure. **C** Biplot of simple RDA. **D** Biplot of partial RDA conditioned on population structure. The arrows represent correlations of the environmental variables with RDA axes that are shown in greater detail in Table S6. Abbreviation in the biplots: Asp aspect, CVR CoefVar_Rain, ET Elevation+Temperature, LRS Latitude+Rain+Solar_rad, Slp Slope, SBD Soil_bulk_density, SCC1 Soil_carbon_content (0–15 cm), SCC2 Soil_carbon_content (30 cm), SpH Soil_pH, SSC Soil_silt_content, SWC Soil_water_capacity, SDT StdDev_Temperature.

To quantify the relative contributions of the environment and space to genomic variation, we performed RDA on SNPs by taking all environmental variables as a whole and incorporating spatial autocorrelation. RDA showed that environmental variables explained 15.12% (${R}_{\rm{adj}}^{2}=0.107$; *p* = 0.0002) of SNP variation while spatial autocorrelation captured by dbMEMs, which are eigenfunctions of a spatial network (Legendre and Legendre 2012, Dray et al. 2006), explained 44.95% (${R}_{\rm{adj}}^{2}=0.285$; *p* = 0.0002; Fig. 4A). We found 10.63% of SNP variation is jointly explained by environmental variables and spatial autocorrelation, and 4.49% (${R}_{\rm{adj}}^{2}=0.013$; *p* = 0.0002) was solely explained by environmental variables (Fig. 4A). Considering the confounding effect between environment and population structure, we treated ancestry coefficients (*K =* 4) as covariates in partial RDA when examining the effect of environmental variables on SNP variation. The partial RDA indicated that population structure explained 15.43% (${R}_{\rm{adj}}^{2}=0.148$; *p* = 0.0002) of SNP variation, and environmental variables solely explained 8.71% (${R}_{\rm{adj}}^{2}=0.048$; *p* = 0.0002) of SNP variation when conditioned on population structure (Fig. 4A).

#### Relative importance of individual environmental variables for SNP variation

After confirming an association between genomic variation and the environment, we further investigated the effects of individual environmental variables. In simple RDA models with separate fitting of each environmental variable, permutation tests showed that all of the 12 environmental variables were significantly associated with SNP variation (*p* < 0.005; Table S5.1). Without constraining on population structure, the synthetic variable ’*Latitude+Rain+Solar_rad*’ (Tables S1 and S2) explained the highest proportion of SNP variation (3.89%; Fig. 4B; Table S5.1). In contrast, in partial RDA models conditioned on population structure, ’*Soil_water_capacity*’ explained the highest proportion of SNP variation (1.18%; Fig. 4B; Table S5.2) whereas the proportion of SNP variation explained by ’*Latitude+Rain+Solar_rad*’ reduced to 0.86%. The variable ’*Aspect*’ presented the lowest but significant association with SNP variation in both simple and partial RDA conditioned on population structure (Table S5.1 and S5.2). The explained variation of ’*Soil_water_capacity*’, ’*CoefVar_Rain*’, which refers to coefficients of variation of precipitation in the growing season, and ’*Aspect*’ decreased less than other environmental variables after conditioned on population structure. This indicates that they correlated less with population structure. We also investigated marginal effects in models that incorporated all environmental variables by considering correlations between environmental variables. The variables ’*Latitude+Rain+Solar_rad*’ and ’*Soil_water_capacity*’ once again showed the highest marginal effect in the simple RDA and partial RDA conditioned on population structure, respectively (Fig. 4B; Table S5.3 and S5.4).

RDA biplots provided further information on the relative importance of environmental gradients. The biplot of the simple RDA (Fig. 4C) showed a population structure consistent with the four genetic clusters identified by *ALStructure*. The first and second RDA axes corresponded to genetic differentiation in the north-south and west-east directions, respectively (Fig. 4C). The first RDA axis was strongly (*r* = 0.911; Table S6) correlated with the variable “*Latitude+Solar_rad*” (Fig. 4C). When conditioned on population structure, two water-related variables, “*Soil_water_capacity*” (SWC; *r* = 0.697) and “*CoefVar_Rain*” (CVR; *r* = −0.662), were the most influential predictors on the first RDA axis (Fig. 4D and Table S6). However, if conditioned on spatial autocorrelation rather than on population structure, the effects of all environmental variables decreased significantly (Fig. S9 and Table S6). This indicated a strong correlation of environmental gradients with spatial autocorrelation.

#### Association of population structure with environment and space

With reference to our hypothesis that the diverse environments in the Southern Levant were an important factor in shaping populations, we quantified the relative contributions of environment and space to population structure (*K* = 4) with RDA on ancestry coefficients. As expected, a high proportion of population structure that was explained by environmental variables (42.91 of 46.59%; the right column of Fig. 4A) could not be separated from the component explained by spatial autocorrelation. Only 3.68% (${R}_{\rm{adj}}^{2}=0.0358$; *p* = 0.0002) of population structure could be solely explained by environments whereas spatial autocorrelation accounted solely for 39.87% (${R}_{\rm{adj}}^{2}=0.374$; *p* = 0.0002) of population structure (Fig. 4A). This result suggested that spatial autocorrelation had a larger effect on population differentiation of wild barley in the Southern Levant than environmental diversity.

### Adaptive candidates and GO enrichment

The association between genomic variation and environment prompted us to perform genome scans to identify putative adaptive loci. Given the large genome size (~5.3 Gb), we first estimated LD decay to assess whether the marker density of the reduced-representation data was sufficient to accurately identify adaptive genes in these scans. We fitted the *loess* model and Hill-Weir formula with 27,147 genome-wide SNPs. We then observed a rapid decay in LD because *r*^2^ values dropped to half of the highest values of 0.377 and 0.454 after pairwise SNP distances of 213 bp and 125 bp, respectively (Fig. S10). Given the large size of the barley genome, this result indicates a possible difficulty in detecting the precise locations of adaptive loci, except for closely linked loci with the current marker density.

**Fig. 5:**
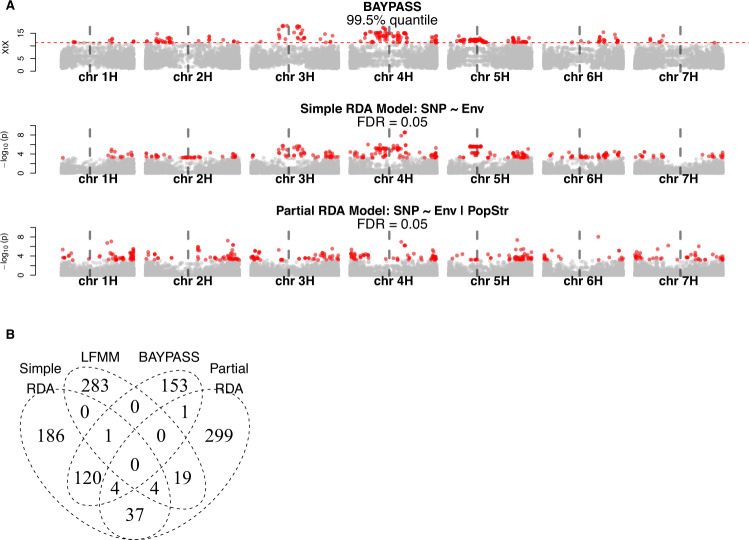
Inference of selection. **A** Genome scans for adaptation signatures. Three Manhattan plots correspond to the *BAYPASS*, simple RDA, and partial RDA conditioned on population structure. Significant SNPs are highlighted as red dots. The positions of centromeres are indicated with vertical gray dash lines. **B** Numbers of significant SNPs detected by four different methods for genome scans.

Three GEA methods, simple RDA, partial RDA, and LFMM, identified 352, 364, and 307 candidate SNPs (FDR < 0.05), respectively, and the outlier method, *BAYPASS*, identified 279 candidate SNPs (*X*^*T*^*X* > 11.05). However, candidate SNPs detected by the four methods hardly overlapped, except simple RDA and *BAYPASS* with 125 common SNPs, 91 of which were located in pericentromeric regions of chromosomes 3H, 4H, and 5H (Fig. 5; File S3). By searching 500 bp adjacent intervals of candidate SNPs, the four methods jointly identified two genes on the chromosome 4H. The first gene *HORVU.MOREX.r2.4HG0308420* locates closely to SNPs associated with the variable ’*Latitude+Rain+Solar_rad*’ in the LFMM analysis (File S4) which encodes an ATP-dependent RNA helicase. The second gene *HORVU.MOREX.r2.4HG0314300* is linked to SNPs associated with ’*Elevation+Temperature*’. It encodes a nucleolar GTP-binding protein (Fig. S11; Table S7; File S4). GO term enrichment analysis identified 2 and 10 enriched GO terms based on candidate SNPs detected by simple RDA and *BAYPASS*, respectively (Table S8). No GO term was enriched based on the results of partial RDA and LFMM.

## Discussion

Our study indicated that geography and spatial autocorrelation were better predictors of genomic variation than environmental gradients even though the diverse environments of the Southern Levant are expected to impose strong natural selection (Hübner et al. 2009; Nevo et al. 1979). These findings imply that genomic variation of wild barley in the Southern Levant was mainly driven by neutral processes consistent with a neutralist perspective (e.g., Volis et al. 2001, 2003, 2005). However, environmental variables were still associated with a relatively small but considerable proportion of genomic variation (15.12%; Fig. 4A), suggesting that natural selection and hitch-hiking may have a detectable effect on the structure of genetic diversity.

### Strong population structure of B1K+ and IPK genebank collections

Three clusters of wild barley from the Levant region that correspond to eco-geographical habitats were previously characterized using SSR markers and an SNP array developed for cultivated barley (Hübner et al. 2012) and morphological traits (Hübner et al. 2013). Our results are consistent with previous findings, except that the previously reported desert cluster (Hübner et al. 2012) was split into two clusters (Fig. 1B–D), which was evident in the *ALStructure* analysis with *K* = 3 and *K =* 4 (Fig. 2). Difference to previous study resulted from an increased marker number but also to the inclusion of additional accessions collected in the Negev Desert in 2011 (File S2). Although genetic clusters were consistent with eco-geographical habitats, caution should be exercised when interpreting the results of model-based methods. First, the number of ancestral populations might be overestimated due to isolation by distance (Bradburd et al. 2018). Second, the high proportion of admixed accessions (174 of 244 B1K+ accessions; 71.3%) might not result from admixture. Both spatial autocorrelation (Bradburd et al. 2018) and demographic history (Lawson et al. 2018) such as bottlenecks that likely occur in self-pollinating species (Hartfield et al. 2017), may lead to high admixture proportions in model-based methods.

The joint PCA incorporating the IPK wild barley collection indicated a strong effect of an unbalanced sample size of accessions from Israel (616 of 1365 accessions) on a PCA (Fig. 3A, B). This comparison highlighted the importance of balanced sampling when analyzing population structure because unequal sample sizes among groups could lead to the distortion of PCs (McVean 2009). The PCA, based on all accessions, compressed the accessions with a broad geographical origin across the whole distribution range of wild barley into a cluster (Fig. 3A) that did not appropriately reflect their wide geographical origin. In contrast, a PCA with a more balanced sample of accessions from the whole species range revealed that the wild barley from the Southern Levant regions contained only a small proportion of the total diversity of wild barley (Fig. 3B). However, the PCA of the complete sample revealed that accessions from Greece and Cyprus clustered with accessions from the Southern Levant (Figs. 3A and S12A). This suggested they originated from the Southern Levant or adjacent areas without a sufficiently long history of differentiation from the ancestral populations. Likewise, 579 IPK accessions of unknown origins might be closely related to the Levant region as they strongly overlapped with our B1K+ population (Fig. S12).

### Evidence for geographical pattern of gene flow

We expected that gene flow among wild barley populations was limited because of a low rate of outcrossing (<2%; Abdel-Ghani et al. 2004), and seed dispersal occurred mainly within 1.2 m (Volis et al. 2010). However, self-fertilizing plants can establish a population with a single seed after long-distance dispersal (Baker, 1967), and the long spiky awns attached to seeds of wild barley facilitates dispersal by zoochory. Over a sufficiently long period, gene flow across landscapes might accumulate via occasional dispersal and outcrossing. *EEMS* (Petkova et al. 2016) was previously used to identify gene flow barriers in plant populations across large geographical ranges, e.g., rice (Gutaker et al. 2020) and spruce (Tsuda et al. 2016). In our data, *EEMS* revealed fine-scale patterns of gene flow attributable to geography, particularly of the Sea of Galilee and the Jordan Valley, which had not been previously identified by inferring gene flow between genetic clusters (Hübner et al. 2012). These geographical separations appear to promote genetic differentiation within a short geographical distance that interfered with isolation-by-distance patterns (Fig. S8B). The analysis of *ResistanceGA*, which accounted for the non-independence of samples, also suggested a stronger effect on genetic differentiation by geographical barriers than by isolation-by-distance (Table S3).

CBI (Lundgren and Ralph 2019) detected trends of gene flow in opposite directions in eastern and western regions (Fig. 1G). This contradicted the net gene flow from north to south identified by Hübner et al. (2012). The different conclusions regarding gene flow directions in western Israel were likely due to the manner in which geographical information was incorporated into the analyses. While Hübner et al. (2012) assigned accessions according to genetic clustering, our assignment emphasized geographical origin. CBI gene flow rates expressed the probability that a population descended from another population per unit time (Lundgren and Ralph 2019). Consequently, a history of recent colonization might explain gene flow trends in our data. Incorporating historical genome recombination to infer gene flow at different time periods might provide a clearer picture (Al-Asadi et al. 2019). Errors in gene flow inference could result from sampling biases, missing and erroneous genotypic values caused by low sequencing depth, and also from uneven distributions of markers due to the nature of GBS (Elshire et al. 2011, Poland et al. 2012). However, imbalanced sampling should not bias our results because *EEMS* and CBI are insensitive to unequal sample numbers (Petkova et al. 2016, Lundgren and Ralph 2019).

### Effects of environment and geographical distance on SNP variation

RDA analysis indicated that environmental gradients explained a substantial portion of SNP variation and population structure (Fig. 4A). This analysis did not include all possible environmental effects because comprehensive environmental data were not available. For example, the adaptive trait *drought stress recovery* is associated with the rainfall predictability in wild barley (Galkin et al. 2018), but such data were only available for some collection sites. In addition, the control for collinearity and nonlinear environmental effects that RDA did not account for, might lead to unexplained genetic variation in our analysis.

Phenotypic studies suggested the importance of rainfall in the evolution of wild barley in the Southern Levant (Hübner et al. 2013, Galkin et al. 2018; Volis et al. 2002a, b; Volis 2011). Our RDA analysis indicated that variables related to water availability (’*Latitude+Rain+Solar_rad*’ and ’*Soil_water_capacity*’) were the most important drivers of genomic variation (Fig. 4B–D; Table S5). It was not possible to specify the effects of individual environmental gradients because they were highly correlated. For example, we could not separate the effect of precipitation from latitude, which is highly relevant for the timing of flowering in barley (Russell et al. 2016). Unlike other environmental variables, ’*Aspect*’ had few confounding effects with other gradients and population structure (Fig. 4B). ’*Aspect*’ was the strongest predictor when conditioned on spatial autocorrelation (Fig. S9; Table S5). In the Southern Levant, south-facing slopes might be more exposed to drought and heat than north-facing slopes due to higher solar radiation, resulting in significantly stronger selection within only a few hundred meters, referred to as the Evolution Canyon model (Nevo et al. 2005, Bedada et al. 2014). Our results suggest that ’*Aspect*’ might reflect a minor but pervasive effect of microclimate in the Southern Levant that could not be represented by climate data at the current resolution. In *Mimulus guttatus*, an important locus of microgeographical adaptation was successfully identified by integrating quantitative trait loci mapping and population genomic analyses (Hendrick et al. 2016). A similar approach might be used to investigate the genetic architecture of adaptation to microclimatic conditions in wild barley.

By using dbMEMs, which model the effects of spatial autocorrelation on SNP variation, our RDA revealed that high proportions of SNP variation (45%) and population structure (83%) were explained by spatial autocorrelation (Fig. 4A). The lower proportion of SNP variation attributed to environments (Fig. 4A) indicated that environmental selection might be an influential but not a dominant driver of genetic differentiation. In contrast to our findings, environment had a significantly stronger effect than geographical distance on diversity in *Boechera stricta* (Lee and Mitchell-Olds 2011). However, in *Arabidopsis thaliana* (Lasky et al. 2012), sorghum (Lasky et al. 2015), rice (Gutaker et al. 2020) and wild tomato (Gibson and Moyle 2020), the contribution of the environment was comparable and highly overlapped with geographical distance. This suggested that isolation-by-distance was a robust and widespread pattern in a small geographical range like in our case and over a large geographical scale (Gutaker et al. 2020; Lasky et al. 2012, 2015; Gibson and Moyle 2020). Complex spatial structures confounding with environmental gradients are a pervasive challenge in the study of local adaptation (Excoffier et al. 2009, de Villemereuil et al. 2014). In particular, population genetic analyses tend to be biased by spatial structure (Battey et al. 2020). For this reason, phenotypic studies using crosses between accessions and common garden experiments are also required to distinguish between genetic variation attributed to local adaptation and spatial autocorrelation. Additionally, we noted that a high percentage of SNP variation (51%; Fig. 4A) remained unexplained even after incorporating dbMEMs. This could be due to either unknown evolutionary forces that are independent of spatial autocorrelation or to the limitations of our current dataset and methodologies.

### Lack of strong evidence to pinpoint adaptive loci

The rapid decay of LD within a few hundred base pairs (Fig. S10) was consistent with similar studies of wild barley populations from the Middle East and Central Asia (Morrell et al. 2005). Reduced-representation sequencing approaches tend to have limited power in identifying adaptive loci, especially for genomes with high levels of recombination (Tiffin and Ross-Ibarra 2014). Rapid LD decay and a large genome size of ~5.3 Gb indicate that the marker density of this study might not allow precise genome scans. To account for this caveat and to control for false-positive rates, we combined the results from multiple methods of genome scans (Forester et al. 2018; Lotterhos and Whitlock 2015; Rellstab et al. 2015). Although different methods detected different signals, we considered only overlapping signals between scans to be promising adaptive signatures because our goal was to identify stress-tolerance loci that could be useful in barley breeding. In particular, the correlation between populations raised a concern of false positives in genome scans (de Villemereuil et al. 2014) as we studied populations from a small geographical range.

Significant polymorphisms hardly overlapped between methods (Fig. 5B). This observation might be explained by (1) lack of adaptive loci with large effects, (2) strong confounding effect of population structure, and (3) limitations of the dataset. Although there was no robust evidence of the identification of adaptation genes, the genome scans based on *X*^*T*^*X* and simple RDA identified significant correlations with environmental variables and strong genetic differentiation in the pericentromeric regions of the chromosome 3H, 4H, and 5H (Fig. 5A). However, these associations were not observed in the partial RDA and LFMM analyses. Although the *X*^*T*^*X* statistics accounted for the covariance of allele frequencies (i.e., population structure) among populations (Günther and Coop 2013), spurious signals of selection might arise if self-fertilization inflated false-positive values via strong genetic drift (Hodgins and Yeaman 2019). For this reason and because of a strong association between population structure and environments (Fig. 4A), false positives were expected for the outlier and GEA methods even with a correction for population structure. In spite of the concern about false positives, the high degree of putative selection-driven differentiation was still remarkable. Similar patterns of genetic differentiation in pericentromeric regions were reported in previous studies of barley (Wang et al. 2018, Fang et al. 2014, Contreras-Moreira et al. 2019), teosinte (Pyhäjärvi et al. 2013) and maize (Navarro et al. 2017). Theoretical studies suggested that adaptation with gene flow could result in divergent linkage groups of locally beneficial alleles in low-recombination regions (Yeaman and Whitlock 2011, Bürger and Akerman 2011, Akerman and Bürger 2014). These conclusions were supported by simulation and empirical studies, e.g., in stickleback, sunflower, and *Arabidopsis lyrata* (Berner and Roesti 2017, Hämälä and Savolainen 2019, Renaut et al. 2013, Samuk et al. 2017). Low-recombination pericentromeric regions of wild barley were reported to have significantly higher ratios of non-synonymous to synonymous substitution (*π*_a_/*π*_s_) than other genomic regions (Baker et al. 2014). This suggested a tendency to accumulate genetic load in pericentromeric regions. Moreover, in terms of conditional neutrality, the accumulation of conditionally deleterious mutations in habitats where they are neutral could lead to genotype-environment interactions of fitness if migration is weak relative to genetic drift (Mee and Yeaman 2019). Taken together, given weak gene flow, high rates of self-fertilization, and variable recombination rates over the genome, a long-term accumulation of conditionally deleterious mutations might result in locally neutral linkage of alleles in low-recombination genomic regions. This could create a pattern of polymorphism that might resemble local adaptation and explain our observations in the pericentromeric regions.

### Conclusion and outlook

We observed a stronger effect of nonselective factors such as geography and isolation-by-distance on total genetic diversity in the wild barley populations of the diverse and stressful environments of the Southern Levant. Nevertheless, natural selection has a small but significant influence on genomic variation. This might be potentially valuable for barley breeding because water availability, i.e., precipitation and soil water capacity, was the most strongly correlated environmental variable. Outlier test and simple RDA identified genomic regions that might contribute to local adaptation, but these regions were not robustly identified by the different tests applied. One limitation of our study was therefore that only a small proportion of the wild barley genome was sequenced by the GBS approach. This was suitable for analyzing genome-wide patterns of variation and mapping of causal genes (Milner et al. 2019), but was not powerful enough for pinpointing genomic targets of local adaptation. In the near future, whole genome sequencing of wild barley accessions (Sato et al. 2021) and the development of a barley pangenome (Jayakodi et al. 2020) will greatly increase the ability of population genomic approaches to understand wild barley adaptation and facilitate the mining of useful alleles for plant breeding. Such approaches can be combined with common garden and transplantation experiments of wild barley genotypes to measure fitness effects in different environments (Hübner et al. 2013, Volis 2011), gene expression studies of differentially adapted genotypes (Hübner et al. 2015) and mapping populations. Given the major impact of isolation-by-distance on genomic variation, adaptive genetic variation was likely confounded with population structure. Mapping populations with sufficient genome recombination evaluated in different environments permitted the disentangling of adaptive and neutral variation, as shown in such populations developed from wild and cultivated barley (Herzig et al. 2018; Wiegmann et al. 2019). Whole-genome resequencing followed by computational analysis can be rationalized to analyze a large number of genotypes such as the complete B1K population. Consequently, we believe that population genomic analysis of differentially adapted crop-wild relatives will complement other approaches to understanding plant adaptation and enable the use of this information for breeding (Bohra et al. 2021).

## Supplementary information

Supplementary text, tables, and figures

File S1

File S2

File S3

File S4

File S5

## Data Availability

The GBS data of B1K+ accessions collected in this study have been archived at the European Nucleotide Archive (ENA) with project ID PRJEB47405. The ENA sample IDs are available in Supplementary File S5. The geographical coordinates and environmental data are available in Supplementary File S1. The R code used for analysis is archived at https://kjschmidlab.gitlab.io/b1k-gbs/.

## References

[CR1] Abdel-Ghani AH, Parzies HK, Omary A, Geiger HH (2004). Estimating the outcrossing rate of barley landraces and wild barley populations collected from ecologically different regions of Jordan. Theor Appl Genet.

[CR2] Akerman A, Bürger R (2014). The consequences of gene flow for local adaptation and differentiation: a two-locus two-deme model. J Math Biol.

[CR3] Al-Asadi H, Petkova D, Stephens M, Novembre J (2019). Estimating recent migration and population-size surfaces. PLoS Genet.

[CR4] Baker HG (1967). Support for Baker’s law-as a rule. Evolution.

[CR5] Baker K, Baker K, Bayer M, Cook N, Dreißig S, Dhillon T, Russell J, Hedley PE, Morris J, Ramsay L, Colas I (2014). The low-recombining pericentromeric region of barley restricts gene diversity and evolution but not gene expression. Plant J.

[CR6] Battey C, Ralph PL, Kern AD (2020). Space is the place: effects of continuous spatial structure on analysis of population genetic data. Genetics.

[CR7] Baum M, Grando S, Backes G, Jahoor A, Sabbagh A, Ceccarelli S (2003). QTLs for agronomic traits in the mediterranean environment identified in recombinant inbred lines of the cross’ Arta’ × *H. spontaneum* 41-1. Theor Appl Genet.

[CR8] Bedada G, Westerbergh A, Nevo E, Korol A, Schmid KJ (2014). DNA sequence variation of wild barley *Hordeum spontaneum* (L.) across environmental gradients in Israel. Heredity.

[CR9] Berner D, Roesti M (2017). Genomics of adaptive divergence with chromosome-scale heterogeneity in crossover rate. Mol Ecol.

[CR10] Bhatia G, Patterson N, Sankararaman S, Price AL (2013). Estimating and interpreting FST: the impact of rare variants. Genome Res.

[CR11] Bohra A, Kilian B, Kilian B, Sivasankar S, Caccamo M, Mba, C, McCouch SR, Varshney RK (2021) Reap the crop wild relatives for breeding future crops. Trends Biotechnol. 10.1016/j.tibtech.2021.08.009 10.1016/j.tibtech.2021.08.009 34629170

[CR12] Bradburd GS, Coop GM, Ralph PL (2018). Inferring continuous and discrete population genetic structure across space. Genetics.

[CR13] Bürger R, Akerman A (2011). The effects of linkage and gene flow on local adaptation: a two-locus continent–island model. Theor Popul Biol.

[CR14] Cabreros I, Storey JD (2019). A likelihood-free estimator of population structure bridging admixture models and principal components analysis. Genetics.

[CR15] Caldwell KS, Russell J, Langridge P, Powell W (2006). Extreme population-dependent linkage disequilibrium detected in an inbreeding plant species, *Hordeum vulgare*. Genetics.

[CR16] Capblancq T, Luu K, Blum MG, Bazin E (2018). Evaluation of redundancy analysis to identify signatures of local adaptation. Mol Ecol Resour.

[CR17] Caye K, Jumentier B, Lepeule J, François O (2019). LFMM 2: fast and accurate inference of gene-environment associations in genome-wide studies. Mol Biol Evol.

[CR18] Contreras-Moreira B, Serrano-Notivoli R, Mohammed NE, Cantalapiedra CP, Beguería S, Casas AM, Igartua E (2019). Genetic association with high-resolution climate data reveals selection footprints in the genomes of barley landraces across the Iberian Peninsula. Mol Ecol.

[CR19] Dawson IK, Russell J, Powell W, Steffenson B, Thomas WTB, Waugh R (2015). Barley: a translational model for adaptation to climate change. New Phytol.

[CR20] Dray S, Legendre P, Peres-Neto PR (2006). Spatial modelling: a comprehensive framework for principal coordinate analysis of neighbour matrices (pcnm). Ecol Model.

[CR21] Dray S, Bauman D, Blanchet G, Borcard D, Clappe S, Guenard G, Jombart T, Larocque G, Legendre P, Madi N, Wagner HH (2019) *adespatial*: multivariate multiscale spatial analysis. R package version 0.3-7. https://CRAN.R-project.org/package=adespatial

[CR22] Elshire RJ, Glaubitz JC, Sun Q, Poland JA, Kawamoto K, Buckler ES, Mitchell SE (2011). A robust, simple genotyping-by-sequencing (GBS) approach for high diversity species. PLoS ONE.

[CR23] Excoffier L, Hofer T, Foll M (2009). Detecting loci under selection in a hierarchically structured population. Heredity.

[CR24] Fang Z, Gonzales AM, Clegg MT, Smith KP, Muehlbauer GJ, Steffenson BJ, Morrell PL (2014). Two genomic regions contribute disproportionately to geographic differentiation in wild barley. G3.

[CR25] Fick SE, Hijmans RJ (2017). Worldclim 2: new 1-km spatial resolution climate surfaces for global land areas. Int J Climatol.

[CR26] Forester BR, Lasky JR, Wagner HH, Urban DL (2018). Comparing methods for detecting multilocus adaptation with multivariate genotype–environment associations. Mol Ecol.

[CR27] Forester BR, Jones MR, Joost S, Landguth EL, Lasky JR (2016). Detecting spatial genetic signatures of local adaptation in heterogeneous landscapes. Mol Ecol.

[CR28] Galkin E, Dalal A, Evenko A, Fridman E, Kan I, Wallach R, Moshelion M (2018). Risk-management strategies and transpiration rates of wild barley in uncertain environments. Physiol Plant.

[CR29] Gautier M (2015). Genome-wide scan for adaptive divergence and association with population-specific covariates. Genetics.

[CR30] Gibson MJ, Moyle LC (2020). Regional differences in the abiotic environment contribute to genomic divergence within a wild tomato species. Mol Ecol.

[CR31] Günther T, Coop G (2013). Robust identification of local adaptation from allele frequencies. Genetics.

[CR32] Gutaker RM, Groen SC, Bellis ES, Choi JY, Pires IS, Bocinsky RK, Slayton ER, Wilkins O, Castillo CC, Negrão S (2020). Genomic history and ecology of the geographic spread of rice. Nat Plants.

[CR33] Hämälä T, Savolainen O (2019). Genomic patterns of local adaptation under gene flow in *arabidopsis lyrata*. Mol Biol Evol.

[CR34] Harlan JR, Zohary D (1966). Distribution of wild wheats and barley. Science.

[CR35] Hartfield M, Bataillon T, Glémin S (2017). The evolutionary interplay between adaptation and self-fertilization. Trends Genet.

[CR36] Hendrick MF, Finseth FR, Mathiasson ME, Palmer KA, Broder EM, Breigenzer P, Fishman L (2016). The genetics of extreme microgeographic adaptation: an integrated approach identifies a major gene underlying leaf trichome divergence in yellowstone mimulus guttatus. Mol Ecol.

[CR37] Hengl T, Mendes de Jesus J, Heuvelink GBM, Ruiperez Gonzalez M, Kilibarda M, Blagotić A, Shangguan W, Wright MN, Geng X, Bauer-Marschallinger B (2017). Soilgrids250m: Global gridded soil information based on machine learning. PLoS ONE.

[CR38] Herzig P, Herzig P, Maurer A, Draba V, Sharma R, Draicchio F, Bull H, Milne L, Thomas WTB, Flavell AJ, Pillen K (2018). Contrasting genetic regulation of plant development in wild barley grown in two European environments revealed by nested association mapping. J Exp Bot.

[CR39] Hill W, Weir B (1988). Variances and covariances of squared linkage disequilibria in finite populations. Theor Popul Biol.

[CR40] Hodgins KA, Yeaman S (2019). Mating system impacts the genetic architecture of adaptation to heterogeneous environments. New Phytol.

[CR41] House GL, Hahn MW (2018). Evaluating methods to visualize patterns of genetic differentiation on a landscape. Mol Ecol Resour.

[CR42] Hübner S, Korol AB, Schmid KJ (2015). Rna-seq analysis identifies genes associated with differential reproductive success under drought-stress in accessions of wild barley hordeum spontaneum. BMC Plant Biol.

[CR43] Hübner S, Bdolach E, Ein-Gedy S, Schmid KJ, Korol A, Fridman E (2013). Phenotypic landscapes: phenological patterns in wild and cultivated barley. J Evol Biol.

[CR44] Hübner S, Günther T, Flavell A, Fridman E, Graner A, Korol A, Schmid KJ (2012). Islands and streams: clusters and gene flow in wild barley populations from the Levant. Mol Ecol.

[CR45] Hübner S, Höffken M, Oren E, Haseneyer G, Stein N, Graner A, Schmid K, Fridman E (2009). Strong correlation of wild barley (*Hordeum spontaneum*) population structure with temperature and precipitation variation. Mol Ecol.

[CR46] Jakob SS, Rödder D, Engler JO, Shaaf S, Özkan H, Blattner FR, Kilian B (2014). Evolutionary history of wild barley (*Hordeum vulgare* subsp. *spontaneum*) analyzed using multilocus sequence data and paleodistribution modeling. Genome Biol Evol.

[CR47] Jayakodi M, Padmarasu S, Haberer G, Bonthala VS, Gundlach H, Monat C, Lux T, Kamal N, Lang D, Himmelbach A, Ens J, Zhang X-Q, Angessa TT, Zhou G, Tan C, Hill C, Wang P, Schreiber M, Boston LB, Plott C, Jenkins J, Guo Y, Fiebig A, Budak H, Xu D, Zhang J, Wang C, Grimwood J, Schmutz J, Guo G, Zhang G, Mochida K, Hirayama T, Sato K, Chalmers KJ, Langridge P, Waugh R, Pozniak CJ, Scholz U, Mayer KFX, Spannagl M, Li C, Mascher M, Stein N (2020). The barley pan-genome reveals the hidden legacy of mutation breeding. Nature.

[CR48] Kawecki TJ, Ebert D (2004). Conceptual issues in local adaptation. Ecol Lett.

[CR49] Kilian B, Özkan H, Kohl J, von Haeseler A, Barale F, Deusch O, Brandolini A, Yucel C, Martin W, Salamini F (2006). Haplotype structure at seven barley genes: relevance to gene pool bottlenecks, phylogeny of ear type and site of barley domestication. Mol Genet Genom.

[CR50] Lasky JR, Des Marais DL, McKAY JK, Richards JH, Juenger TE, Keitt TH (2012). Characterizing genomic variation of *arabidopsis thaliana*: the roles of geography and climate. Mol Ecol.

[CR51] Lasky JR, Upadhyaya HD, Ramu P, Deshpande S, Hash CT, Bonnette J, Juenger TE, Hyma K, Acharya C, Mitchell SE (2015). Genome-environment associations in sorghum landraces predict adaptive traits. Sci Adv.

[CR52] Lawson DJ, Van Dorp L, Falush D (2018). A tutorial on how not to over-interpret STRUCTURE and ADMIXTURE bar plots. Nat Commun.

[CR53] Lee C-R, Mitchell-Olds T (2011). Quantifying effects of environmental and geographical factors on patterns of genetic differentiation. Mol Ecol.

[CR54] Leek JT (2011). Asymptotic conditional singular value decomposition for high-dimensional genomic data. Biometrics.

[CR55] Legendre P, Legendre L (2012) Canonical analysis. In: Numerical ecology, 3rd English edn, chap. 11. Elsevier Science BV, The Netherlands, pp 625–710

[CR56] López-Goldar X, Agrawal AA (2021). Ecological interactions, environmental gradients, and gene flow in local adaptation. Trends Plant Sci.

[CR57] Lotterhos KE, Whitlock MC (2015). The relative power of genome scans to detect local adaptation depends on sampling design and statistical method. Mol Ecol.

[CR58] Lundgren E, Ralph PL (2019). Are populations like a circuit? Comparing isolation by resistance to a new coalescent-based method. Mol Ecol Resour.

[CR59] Makowski D, Ben-Shachar M, Lüdecke D (2019). bayestestR: describing effects and their uncertainty, existence and significance within the Bayesian framework. J Open Source Softw.

[CR60] Mascher M, Gundlach H, Himmelbach A, Beier S, Twardziok SO, Wicker T, Radchuk V, Dockter C, Hedley PE, Russell J (2017). A chromosome conformation capture ordered sequence of the barley genome. Nature.

[CR61] Mascher M (2019) Pseudomolecules and annotation of the second version of the reference genome sequence assembly of barley cv. morex [morex v2]. https://doi.ipk-gatersleben.de:443/DOI/83e8e186-dc4b-47f7-a820-28ad37cb176b/d1067eba-1d08-42e2-85ec-66bfd5112cd8/2

[CR62] McVean G (2009) A genealogical interpretation of principal components analysis. PLoS Genet 5(10):e1000686 10.1371/journal.pgen.1000686 PMC2757795 19834557

[CR63] Mee JA, Yeaman S (2019). Unpacking conditional neutrality: genomic signatures of selection on conditionally beneficial and conditionally deleterious mutations. Am Nat.

[CR64] Milner SG, Jost M, Taketa S, Mazón ER, Himmelbach A, Oppermann M, Weise S, Knüpffer H, Basterrechea M, König P (2019). Genebank genomics highlights the diversity of a global barley collection. Nat Genet.

[CR65] Morrell PL, Toleno DM, Lundy KE, Clegg MT (2005). Low levels of linkage disequilibrium in wild barley (*Hordeum vulgare* ssp. *spontaneum*) despite high rates of self-fertilization. Proc Natl Acad Sci USA.

[CR66] Navarro JAR, Willcox M, Burgueño J, Romay C, Swarts K, Trachsel S, Preciado E, Terron A, Delgado HV, Vidal V (2017). A study of allelic diversity underlying flowering-time adaptation in maize landraces. Nat Genet.

[CR67] Nevo E, Zohary D, Brown A, Haber M (1979). Genetic diversity and environmental associations of wild barley, *Hordeum spontaneum*, in Israel. Evolution.

[CR68] Nevo E, Beharav A, Meyer RC, Hackett CA, Forster BP, Russell JR, Powell W (2005). Genomic microsatellite adaptive divergence of wild barley by microclimatic stress in ‘Evolution Canyon’, Israel. Biol J Linn Soc.

[CR69] Oksanen J, Blanchet FG, Friendly M, Kindt R, Legendre P, McGlinn D, Minchin PR, O’Hara RB, Simpson GL, Solymos P, Stevens MHH, Szoecs E, Wagner H (2019) *vegan*: community ecology package. R package version 2.5-6. https://CRAN.R-project.org/package=vegan

[CR70] Pankin A, Altmüller J, Becker C, von Korff M (2018). Targeted resequencing reveals genomic signatures of barley domestication. New Phytol.

[CR71] Pekel J-F, Cottam A, Gorelick N, Belward AS (2016). High-resolution mapping of global surface water and its long-term changes. Nature.

[CR72] Pembleton L, Cogan N, Forster J (2013). StAMPP: an R package for calculation of genetic differentiation and structure of mixed-ploidy level populations. Mol Ecol Res.

[CR73] Peterman WE (2018). Resistancega: an r package for the optimization of resistance surfaces using genetic algorithms. Methods Ecol Evol.

[CR74] Petkova D, Novembre J, Stephens M (2016). Visualizing spatial population structure with estimated effective migration surfaces. Nat Genet.

[CR75] Pham A-T, Maurer A, Pillen K, Brien C, Dowling K, Berger B, Eglinton JK, March TJ (2019). Genome-wide association of barley plant growth under drought stress using a nested association mapping population. BMC Plant Biol.

[CR76] Poland JA, Brown PJ, Sorrells ME, Jannink J-L (2012). Development of high-density genetic maps for barley and wheat using a novel two-enzyme genotyping-by-sequencing approach. PLoS ONE.

[CR77] Pyhäjärvi T, Hufford MB, Mezmouk S, Ross-Ibarra J (2013). Complex patterns of local adaptation in teosinte. Genome Biol Evol.

[CR78] Rellstab C, Gugerli F, Eckert AJ, Hancock AM, Holderegger R (2015). A practical guide to environmental association analysis in landscape genomics. Mol Ecol.

[CR79] Renaut S, Grassa CJ, Yeaman S, Moyers BT, Lai Z, Kane NC, Bowers JE, Burke JM, Rieseberg LH (2013). Genomic islands of divergence are not affected by geography of speciation in sunflowers. Nat Commun.

[CR80] Russell J, Mascher M, Dawson IK, Kyriakidis S, Calixto C, Freund F, Bayer M, Milne I, Marshall-Griffiths T, Heinen S (2016). Exome sequencing of geographically diverse barley landraces and wild relatives gives insights into environmental adaptation. Nat Genet.

[CR81] Samuk K, Samuk K, Owens GL, Delmore KE, Miller SE, Rennison DJ, Schluter D (2017). Gene flow and selection interact to promote adaptive divergence in regions of low recombination. Mol Ecol.

[CR82] Sato K, Mascher M, Himmelbach A, Haberer G, Spannagl M, Stein N (2021). Chromosome-scale assembly of wild barley accession ‘OUH602’. G3.

[CR83] Schmid K, Kilian B. Russell J (2018) Barley domestication, adaptation and population genomics. In: The Barley Genome, Springer International Publishing: Cham, pp 317–336

[CR84] Szkiba D, Kapun M, von Haeseler A, Gallach M (2014). SNP2GO: functional analysis of genome-wide association studies. Genetics.

[CR85] Terrazas RA, Balbirnie-Cumming K, Morris J, Hedley PE, Russell J, Paterson E, Baggs EM, Fridman E, Bulgarelli D (2020). A footprint of plant eco-geographic adaptation on the composition of the barley rhizosphere bacterial microbiota. Sci Rep.

[CR86] Tiffin P, Ross-Ibarra J (2014). Advances and limits of using population genetics to understand local adaptation. Trends Ecol Evol.

[CR87] Tsuda Y, Chen J, Stocks M, Källman T, Sønstebø JH, Parducci L, Semerikov V, Sperisen C, Politov D, Ronkainen T (2016). The extent and meaning of hybridization and introgression between siberian spruce (*picea obovata*) and norway spruce (*picea abies*): cryptic refugia as stepping stones to the west?. Mol Ecol.

[CR88] Turner-Hissong SD, Mabry ME, Beissinger TM, Ross-Ibarra J, Pires JC (2020). Evolutionary insights into plant breeding. Curr Opin Plant Biol.

[CR89] de Villemereuil P, Frichot É, Bazin É, François O, Gaggiotti OE (2014). Genome scan methods against more complex models: when and how much should we trust them?. Mol Ecol.

[CR90] Volis S (2011). Adaptive genetic differentiation in a predominantly self-pollinating species analyzed by transplanting into natural environment, crossbreeding and QST-FST test. New Phytol.

[CR91] Volis S, Mendlinger S, Ward D (2002). Differentiation in populations of *Hordeum spontaneum* along a gradient of environmental productivity and predictability: life history and local adaptation. Biol J Linn Soc.

[CR92] Volis S, Mendlinger S, Ward D (2002). Adaptive traits of wild barley plants of Mediterranean and desert origin. Oecologia.

[CR93] Volis S, Zaretsky M, Shulgina I (2010). Fine-scale spatial genetic structure in a predominantly selfing plant: role of seed and pollen dispersal. Heredity.

[CR94] Volis S, Shulgina I, Ward D, Mendlinger S (2003). Regional subdivision in wild barley allozyme variation: adaptive or neutral?. J Hered.

[CR95] Volis S, Verhoeven K, Mendlinger S, Ward D (2004). Phenotypic selection and regulation of reproduction in different environments in wild barley. J Evol Biol.

[CR96] Volis S, Yakubov B, Shulgina I, Ward D, Mendlinger S (2005). Distinguishing adaptive from nonadaptive genetic differentiation: comparison of q st and f st at two spatial scales. Heredity.

[CR97] Volis S, Yakubov B, Shulgina I, Ward D, Zur V, Mendlinger S (2001). Tests for adaptive RAPD variation in population genetic structure of wild barley, *Hordeum spontaneum* Koch. Biol J Linn Soc.

[CR98] Wang X, Chen Z-H, Yang C, Zhang X, Jin G, Chen G, Wang Y, Holford P, Nevo E, Zhang G (2018). Genomic adaptation to drought in wild barley is driven by edaphic natural selection at the Tabigha Evolution Slope. Proc Natl Acad Sci USA.

[CR99] Wiegmann M, Wiegmann M, Maurer A, Pham A, March TJ, Al-Abdallat A, Thomas WTB, Bull HJ, Shahid M, Eglinton J, Baum M, Flavell AJ, Tester M, Pillen K (2019). Barley yield formation under abiotic stress depends on the interplay between flowering time genes and environmental cues. Sci Rep.

[CR100] Yeaman S, Whitlock MC (2011). The genetic architecture of adaptation under migration–selection balance. Evolution.

[CR101] Zheng X, Levine D, Shen J, Gogarten S, Laurie C, Weir B (2012). A high-performance computing toolset for relatedness and principal component analysis of snp data. Bioinformatics.

