# Supplementary material for: Physical geography, isolation by distance and environmental variables shape genomic variation of wild barley (Hordeum vulgare L. ssp. spontaneum) in the Southern Levant
Source: Heredity (Edinb). 2022 Jan 11;128(2):107–19. doi: 10.1038/s41437-021-00494-x (PMC8814169; doi:10.1038/s41437-021-00494-x)
Supplement: Supplementary file 1 — Supplementary text, tables, and figures [file 41437_2021_494_MOESM1_ESM.pdf]

Supplementary Material

Physical geography, isolation by distance and environmental variables shape genomic variation of wild barley (*Hordeum vulgare* L. ssp. *spontaneum*) in the Southern Levant

Che-Wei Chang[1], Eyal Fridman[2], Martin Mascher[3], Axel Himmelbach[3], and Karl Schmid[1]

[1] *University of Hohenheim, Stuttgart, Germany*
[2] *Plant Sciences Institute, Agricultural Research Organization (ARO), The Volcani Center, Israel*
[3] *Leibniz Institute of Plant Genetics and Crop Plant Research (IPK), Gatersleben, Germany*

Genotypic data filtration and processing

Six accessions are included in both the IPK (Milner et al., 2019) and B1K collections (Hübner et al., 2009). We considered the 6 overlapping accessions as part of B1K collection for this study, therefore no accessions overlap between the 244 B1K+ accessions and 1,121 IPK's accessions in this context.

Identification of single nucleotide polymorphism (SNP) was carried out with the same pipeline as in Milner et al. (2019) based on the barley 'Morex V2' assembly. The raw VCF file was first filtered by using an AWK script (bitbucket.org/ipk_dg_public/vcf_filtering) to remove loci with QUAL (mapping quality) less than 40 and genotypic calls with DP (read depth) less than 2. SNPs with a missing proportion higher than 0.2 and accessions with a missing proportion higher than 0.3 were excluded. Next, genotypic data of duplicates were merged according to the following rules: (1) retain a genotypic value if genotypes of duplicates are identical at a given SNP locus, (2) turn a given SNP locus to missing value if any genotype of duplicate conflicts with another, and (3) retain the genotypic value if only one duplicate is not missing at a given SNP locus. Considering that wild barley is predominantly self-fertilizing (Brown et al., 1978), accessions with more than 0.05 heterozygosity among all polymorphic sites were discarded, and then remaining heterozygotes in the dataset were treated as missing values. To investigate the extent of B1K+ accessions covering the genetic variation of the wild barley collection of the IPK genebank, a dataset consisting of 1,121 IPK accessions and 244 B1K+ accessions with 4,793 geographically diverse SNPs was extracted. Briefly, SNPs with a missing proportion $<$0.2 across the whole panel and minor allele frequency (MAF) $>$0.05 among 72 geographically diverse accessions, collected from 13 countries (Russell et al., 2016), were selected. Then, the selected SNPs were pruned by using *PLINK 1.9* (Purcell et al., 2007) to remove SNPs in linkage-disequilibrium (LD) with an r^2 threshold of 0.1, a window size of 50, and a step size of 5, and eventually resulted in 4,793 SNPs.

For analyses of wild barley from the southern Levant, we first extracted 58,616 SNPs with MAF higher than 0.01 and a missing proportion lower than 0.1 among 244 B1K+ accessions. For population structure analysis, LD pruning was performed to exclude redundant markers with an r^2 threshold of 0.1 using *PLINK 1.9* (Purcell et al., 2007) by considering the model assumption of our downstream analysis (Cabreros and Storey, 2019). LD-pruning reduced the unimputed 58,616 SNPs to 19,601 SNPs. For redundancy analysis (RDA), another dataset with no missing values was prepared because RDA requires a non-missing dataset. The whole wild barley panel, including the B1K+ and IPK collections, was imputed by using *BEAGLE 5.1* (Browning et al., 2018)

with default parameters. Next, the 244 B1K+ accessions with the same 58,616 SNPs mentioned above, which have the original missing proportion lower than 0.1 among 244 accessions, were extracted. Lastly, SNPs with MAF lower than 0.05 among 244 B1K+ accessions were removed, resulting in 27,147 imputed SNPs. The imputed SNP data were coded as 0, 1, and 2 according to the counts of alternative alleles and treated as the response variable for RDA. In addition to the RDA, the imputed dataset with 27,147 SNPs was also applied to other genome scan analyses.

Construction of synthetic environmental variables and variable selection

The climatic data including temperature, precipitation and solar radiation was downloaded from *WorldClim2* (Fick and Hijmans, 2017) with the resolution of 30 arc-seconds ($\sim$1 km). The climatic data for growing period of wild barley in Israel, typically between late October and April, was extracted to calculate the bioclimatic variables. Soil property data including a total of 11 variables of four soil layers (0 cm, 5 cm, 15 cm, and 30 cm) with a resolution of 250 m was obtained from SoilGrids (*soilgrids.org*;Hengl et al. 2017). Elevation data with the resolution of 90 m was downloaded from the SRTM database (*https://srtm.csi.cgiar.org/*). Aspects and slopes were calculated based on the elevation data using the *terrain* function in the R package *raster*. Radians of aspects were further converted to cosine values, denoting north-facing and south-facing slopes as 1 and -1, respectively. Environmental data for 244 B1K+ accessions was extracted with the function *extract* in the R package *raster* according to the geographic coordinates of their collecting locations.

To resolve the problem with collinearity, we first combined the highly correlated environmental variables to generate synthetic environmental variables. A synthetic environmental variable was defined as the first principal component scores of a group of highly correlated environmental variables. To cluster the highly correlated environmental variables that can be combined into a synthetic environmental variable, we first grouped all environmental variables by using the complete-linkage clustering based on the dissimilarity between environmental variables. The dissimilarity of a pair of environmental variables was calculated as $1 - R^2$. The R^2 represents the coefficient of determination of two environmental variables. With the dendrogram of the complete-linkage clustering, we could have various clustering combinations by cutting the tree at different levels. Next, we searched the optimal clustering combinations along the dendrogram by using a customized index. The index was calculated as

$$\frac{\sum_{n=1}^{k} SS_{PC1,n}}{SS_{Total} - \sum_{n=1}^{k} SS_{PC1,n}}$$

where *k* is the number of clusters with more than one environmental variable when cutting the dendrogram at a given level. The $SS_{PC1,n}$ and SS_{Total} terms represent the sum of squares of the first principal component calculated from n-th cluster of standardized environmental variables and the total sum of squares of all standardized environmental variables respectively. The $SS_{PC1,n}$ and SS_{Total} terms were calculated by using the *svd* function in R. This index was designed by regarding the sum of squares as the amount of information. Thus, the index can be interpreted as the ratio of information captured by the first PCs of grouped variables to the total information of the original data. The decrease of this index can suggest the loss of information when using the first PCs to represent the grouped variables. In other words, by maximizing the index, we could supposedly find clustering combinations that produced the most informative synthetic environmental

variables to represent highly correlated environmental variables. The optimal cuttoff $1 - R^2$ cutoff was searched between 0 and 1 (Figure S3A). Subsequently, the first PCs, or synthetic environmental variables, were computed according to the optimal clustering combination (Figure S3B). After that, the synthetic environmental variables and the non-synthetic environmental variables were further selected until variance inflation factors (VIFs) of all variables were less than 5. The selection was done by sequentially removing the environmental variable with the highest VIF, but we manually kept the variable related to the accumulated precipitation and the mean temperature ('Latitude+Rain+Solar_rad' and 'Elevation+Temperature') because the precipitation and temperature have been suggested as the most important gradients (Hübner et al., 2009). The VIFs were calculated as $\frac{1}{1 - R_j^2}$, where the R_j^2 is the coefficient of determination obtained by regressing variable j on all the other variables (Legendre and Legendre, 2012). The procedure resulted in 12 environmental variables, including 7 synthetic environmental variables and 5 non-synthetic environmental variables (Table S1). The rotations used to generate synthetic variables are shown in Table S2.

Classification of barrier and non-barrier pixel

To test the hypothesis that geographical barriers are significantly associated with the lower migration rate, we performed a Wilcoxon test by using migration rates estimated by *EEMS* and the geographical barriers defined according to geographical elevation. We assumed the relatively low migration areas on the landscape result from a drastic change in elevation, such as valleys and mountains, therefore we identified geographical barriers by selecting the map pixels with elevation deviating from the majority of pixels (Figure S4). Specifically, we selected the pixels with the top 25%, a cut-off where pixel counts drastically decreased (Figure S4 A), of the absolute values of the standardized elevation and treated selected pixels as the geographical barriers (Figure S4 B). Thereby, pixels of the geographical map were classified as barrier pixels and non-barrier pixels, and the corresponding migration rate of each pixel was extracted from the result of *EEMS*. Finally, a Wilcoxon test was carried out to test if the migration rates are lower at the barrier pixels than the non-barrier pixels.

Supplementary Tables

Table S1 Description of environmental variables used in redundancy analysis (RDA) and genome-environment association (GEA) analysis

Environmental variable	Description	Proportion of explained variance[a]
Aspect	Geographical aspect	-
CoefVar_Rain	Coefficient of variation of precipitation of growing season	-
Elevation+Temperature	First PC of elevation, mean temperature, maximal temperature, and minimal temperature of growing season	0.931
Latitude+Rain+Solar_rad	First PC of latitude, accumulated precipitation, and mean solar radiation of growing season	0.910
Slope	Geographical slope	-
Soil_bulk_density	First PC of soil bulk density of the depths of 0 cm and 5 cm	0.947
Soil_carbon_content(0-15cm)	First PC of soil organic carbon content of the depth of 0 cm, 5 cm, and 15 cm	0.883
Soil_carbon_content(30cm)	Soil organic carbon content of the depth of 30 cm	-
Soil_pH	First PC of soil pH of the depth of 0 cm, 5 cm, 15 cm, and 30 cm	0.969
Soil_silt_content	First PC of silt content (2–50 micro meter) mass fraction in percentage of the depth of 0 cm, 5 cm, 15 cm, and 30 cm	0.970
Soil_water_capacity	First PC of soil water capacity with with FC = pF 2.0, 2.3, and 2.5 until wilt point of the depth of 0 cm	0.962
StdDev_Temperature	Standard deviation of temperature of growing season	-

[a]Proportion of variance explained by the first principal component (PC) of the environmental variables used to generate the synthetic environmental variable. The rotations used to produce first PCs are shown in Table S2.

Table S2 Rotations of first principal component (PC1) used to generate the synthetic environmental variables

Environmental Variable	Rotation of PC1
Aspect	-
CoefVar_Rain	-
Elevation+Temperature	$0.508 \times Elevation - 0.513 \times avg\_temp - 0.491 \times max\_temp - 0.487 \times min\_temp$
Latitude+Rain+Solar_rad	$0.588 \times Latitude + 0.557 \times sum\_prec - 0.587 \times avg\_srad$
Slope	-
Soil_bulk_density	$-0.707 \times sbd(0cm) - 0.707 \times sbd(5cm)$
Soil_carbon_content(0-15cm)	$0.557 \times socc(0cm) + 0.590 \times socc(5cm) + 0.585 \times socc(15cm)$
Soil_carbon_content(30cm)	-
Soil_pH	$0.495 \times pH(0cm) + 0.502 \times pH(5cm) + 0.503 \times pH(15cm) + 0.500 \times pH(30cm)$
Soil_silt_content	$-0.499 \times sltc(0cm) - 0.502 \times sltc(5cm) - 0.503 \times sltc(15cm) - 0.495 \times sltc(30cm)$
Soil_water_capacity	$-0.575 \times swc(pF2.0; 0cm) - 0.582 \times \times swc(pF2.3; 0cm) - 0.575 \times \times swc(pF2.5; 0cm)$
StdDev_Temperature	-

sum_prec: Accumulated precipitation of growing season
avg_srad: Average solar radiation of growing season
avg_temp: Average temperature of growing season
max_temp: Maximum temperature of growing season
min_temp: Minimum temperature of growing season
bd: Soil bulk density (depth of soil layer is shown in the parenthesis)
swc: Soil water capacity (field capacity, referred to pF, and depth of soil layer are shown in the parenthesis)
$socc$: Soil organic carbon content (depth of soil layer is shown in the parenthesis)
pH: Soil pH value (depth of soil layer is shown in the parenthesis)
$sltc$: Silt content (depth of soil layer is shown in the parenthesis)

Table S3 Bootstrap analysis of *ResistanceGA* with 1,000 iterations using random resampling of 75% of sampled populations. The leftmost column shows resistance surfaces used in linear mixed effect models. "Avg.AIC" is the average Akaike information criterion. "Avg.R_m^2" is the average marginal R^2. "Avg.LL" is the average log-likelihood. "n" and "Percent top" is the number and percentage of iterations that a model is selected as the best-supported model. "k" is the number of parameters fitted in a model.

Table S3.1 Bootstrapping with model ranking based on R_m^2

Surface	Avg.AIC	Avg.R_m^2	Avg.LL	n	Percent top	k
Elev+Slope	-5725.286	0.5083	2869.643	1000	100	7
Elev+Slope+Water	-5734.975	0.4414	2876.487	0	0	9
Slope+Water	-5736.288	0.3311	2874.144	0	0	6
Elev	-5741.162	0.3159	2874.581	0	0	4
Water	-5783.527	0.3042	2894.764	0	0	3
Elev+Water	-5739.697	0.3010	2875.849	0	0	6
Distance	-5753.420	0.2113	2878.710	0	0	2
Slope	-5730.958	0.2074	2869.479	0	0	4

Table S3.2 Bootstrapping with model ranking based on AIC

Surface	Avg.AIC	Avg.R_m^2	Avg.LL	n	Percent top	k
Water	-5783.397	0.3055	2894.698	968	96.8	3
Distance	-5752.230	0.2115	2878.115	9	0.9	2
Elev	-5739.792	0.3169	2873.896	7	0.7	4
Elev+Water	-5738.073	0.3014	2875.036	12	1.2	6
Elev+Slope+Water	-5732.737	0.4412	2875.369	0	0.0	9
Slope+Water	-5733.853	0.3317	2872.926	4	0.4	6
Slope	-5729.806	0.2077	2868.903	0	0.0	4
Elev+Slope	-5723.318	0.5080	2868.659	0	0.0	7

Table S4 Gene flow rate and coalescence rate of 10 geographical clusters inferred by coalescence-based method. The rows and columns respectively indicate the source and sink of gene flow. The coalescence rates are shown in diagonal. The 95% credible intervals are shown in the parentheses.

	To									
From	A	B	C	D	E	F	G	H	I	J
A	0.9304 (0-2.04)	1.1481 (0-2.99)	1.3852 (0-4.46)	2.6973 (1.1-4.69)	-	-	-	-	-	-
B	0.66 (0-2.27)	1.2995 (0.42-2.81)	0.7353 (0-2.73)	-	0.7096 (0-2.15)	0.2949 (0-0.89)	-	-	-	-
C	1.2967 (0-3.99)	0.7587 (0-2.65)	0.9776 (0.28-1.96)	0.5604 (0-1.71)	0.3461 (0-1.29)	0.5628 (0-1.29)	-	-	-	-
D	0.4697 (0-1.84)	-	0.4442 (0-1.61)	1.3294 (0.42-2.64)	0.4024 (0-1.62)	2.9335 (1.49-4.88)	-	-	-	-
E	-	1.4597 (0-3.5)	0.3548 (0-1.42)	0.3587 (0-1.32)	0.4086 (0-1)	0.8163 (0-1.91)	0.5671 (0-1.81)	0.7746 (0-2.32)	0.5595 (0-1.71)	-
F	-	0.534 (0-1.6)	0.4686 (0-1.59)	0.6024 (0-2.35)	0.4509 (0-1.89)	2.3576 (0.88-4.33)	0.2834 (0-0.83)	0.5712 (0-1.8)	2.0077 (0.84-3.55)	-
G	-	-	-	-	0.4679 (0-1.39)	0.1673 (0-0.56)	1.2041 (0.4-2.2)	1.1336 (0-3.12)	-	0.0865 (0-0.36)
H	-	-	-	-	3.2381 (0.51-6.32)	1.1729 (0-2.36)	2.0253 (0-3.92)	1.7529 (0.61-3.27)	0.0983 (0-0.41)	0.0655 (0-0.3)
I	-	-	-	-	0.154 (0-0.54)	0.242 (0-0.8)	-	0.1216 (0-0.4)	0.5207 (0.18-1.14)	0.253 (0-1.16)
J	-	-	-	-	-	-	0.1044 (0-0.46)	0.5311 (0.04-0.91)	0.538 (0-0.99)	0.6713 (0.2-1.19)

Table S5 Effect of environmental variables estimated by RDA models. Environmental variables are ordered according to the explained variation. P-values are computed by permutation tests with 5,000 iterations.

Table S5.1	Environmental variable	F	Var	Var (%)	p-value
Individual effect of environmental variables	Latitude+Rain+Solar_rad	9.8046	553.6372	3.8937	0.0002
	Soil_carbon_content(0-15cm)	6.5739	376.0355	2.6447	0.0002
	Soil_carbon_content(30cm)	5.3230	306.0195	2.1522	0.0002
	Soil_silt_content	5.1947	298.7987	2.1015	0.0002
	Soil_bulk_density	5.1081	293.9198	2.0671	0.0002
	Soil_pH	4.8677	280.3609	1.9718	0.0002
	Elevation+Temperature	3.9666	229.2979	1.6127	0.0002
	Soil_water_capacity	3.8998	225.4961	1.5859	0.0002
	StdDev_Temperature	3.5253	204.1527	1.4358	0.0002
	CoefVar_Rain	3.2164	186.4980	1.3116	0.0002
	Slope	2.9324	170.2299	1.1972	0.0002
	Aspect	1.8359	107.0534	0.7529	0.0002

Table S5.2	Environmental variable	F	Var	Var (%)	p-value
Individual effect of environmental variables conditioned on population structure	Soil_water_capacity	3.3934	168.3390	1.1839	0.0002
	CoefVar_Rain	3.1455	156.2026	1.0986	0.0002
	Elevation+Temperature	2.8432	141.3632	0.9942	0.0002
	Soil_pH	2.7389	136.2396	0.9582	0.0002
	Latitude+Rain+Solar_rad	2.4531	122.1676	0.8592	0.0002
	StdDev_Temperature	2.2437	111.8356	0.7865	0.0002
	Soil_carbon_content(0-15cm)	2.2237	110.8457	0.7796	0.0002
	Soil_bulk_density	1.9666	98.1368	0.6902	0.0002
	Soil_carbon_content(30cm)	1.8504	92.3804	0.6497	0.0002
	Slope	1.8143	90.5932	0.6371	0.0002
	Soil_silt_content	1.7954	89.6590	0.6306	0.0002
	Aspect	1.6250	81.2028	0.5711	0.0002

Table S5.3	Environmental variable	F	Var	Var (%)	p-value
Marginal effect of environmental variables	Latitude+Rain+Solar_rad	3.3906	177.1453	1.2459	0.0002
	Soil_water_capacity	3.2845	171.6034	1.2069	0.0002
	StdDev_Temperature	3.2423	169.3995	1.1914	0.0002
	Elevation+Temperature	3.1535	164.7580	1.1587	0.0002
	Soil_carbon_content(0-15cm)	2.4493	127.9675	0.9000	0.0002
	Soil_pH	2.2410	117.0829	0.8234	0.0002
	Soil_bulk_density	2.1565	112.6705	0.7924	0.0002
	Soil_silt_content	2.1446	112.0493	0.7880	0.0004
	CoefVar_Rain	1.9776	103.3233	0.7267	0.0002
	Aspect	1.6364	85.4935	0.6013	0.0018
	Slope	1.5674	81.8895	0.5759	0.0024
	Soil_carbon_content(30cm)	1.5571	81.3511	0.5721	0.0054

Table S5.4	Environmental variable	F	Var	Var (%)	p-value
Marginal effect of environmental variables conditioned on population structure	Soil_water_capacity	2.8405	134.3772	0.9451	0.0002
	Elevation+Temperature	2.4234	114.6438	0.8063	0.0002
	CoefVar_Rain	2.1510	101.7564	0.7157	0.0002
	StdDev_Temperature	2.1193	100.2602	0.7051	0.0002
	Soil_pH	2.0162	95.3822	0.6708	0.0006
	Soil_bulk_density	1.8989	89.8333	0.6318	0.0006
	Latitude+Rain+Solar_rad	1.8864	89.2404	0.6276	0.0002
	Soil_silt_content	1.8743	88.6696	0.6236	0.0004
	Soil_carbon_content(0-15cm)	1.7986	85.0871	0.5984	0.0006
	Soil_carbon_content(30cm)	1.6314	77.1770	0.5428	0.0034
	Slope	1.4805	70.0406	0.4926	0.0080
	Aspect	1.4777	69.9055	0.4916	0.0090

Table S6 Correlation of environmental variables with fitted site scores on first four RDA axes. The correlations were evaluated based on the RDA models (1) without covariates (Simple RDA), (2) conditioned on spatial autocorrelation with dbMEMs (Partial RDA dbMEM), and (3) conditioned on population structure with ancestry coefficients (Partial RDA PopStr)

	Simple RDA				Partial RDA dbMEM				Partial RDA PopStr			
	RDA1	RDA2	RDA3	RDA4	RDA1	RDA2	RDA3	RDA4	RDA1	RDA2	RDA3	RDA4
Aspect	0.067	0.185	0.251	0.3	0.274	-0.365	-0.121	0.237	0.269	0.193	0.072	0.038
CoefVar_Rain	-0.015	0.126	0.543	-0.604	0.013	0.018	0.017	0.008	-0.662	0.035	0.106	0.584
Elevation+Temperature	-0.077	0.33	-0.751	-0.193	0.001	-0.011	0.008	0.004	0.353	-0.557	0.265	-0.046
Latitude+Rain+Solar_rad	0.911	-0.201	0.023	-0.197	-0.004	-0.007	0.005	0.002	0.065	-0.475	-0.049	0.027
Slope	0.335	-0.264	-0.027	-0.178	0.038	-0.065	0.106	-0.069	-0.146	-0.049	0.371	-0.198
Soil_bulk_density	0.59	-0.03	0.037	-0.44	0.041	-0.036	0.021	0.044	-0.272	-0.243	0.176	-0.045
Soil_carbon_content(0-15cm)	0.706	0.157	0.153	-0.218	-0.019	0.022	-0.020	-0.001	0.084	-0.396	0.107	-0.21
Soil_carbon_content(30cm)	0.582	-0.325	0.209	0.221	0.086	0.044	-0.015	0.029	0.169	-0.118	-0.23	-0.254
Soil_pH	-0.526	-0.078	-0.224	0.326	0.021	0.077	0.025	0.079	0.028	0.615	0.354	-0.144
Soil_silt_content	-0.377	0.687	0.203	-0.238	0.149	-0.095	0.027	-0.078	-0.093	0.049	0.12	0.382
Soil_water_capacity	0.325	-0.039	-0.254	0.845	-0.016	-0.004	-0.042	-0.053	0.697	0.44	-0.18	-0.072
StdDev_Temperature	-0.257	-0.453	-0.113	0.216	-0.020	-0.038	-0.006	-0.003	0.118	-0.272	-0.544	-0.267

Table S7 Genes within 500 bp upstream or downstream of the candidate SNPs jointly detected by four methods

Gene	Chr.	Start (bp)	End (bp)	Position of candidate SNP (bp)	Method	Environmental variable	Gene annotation
HORVU.MOREX.r2.4HG0308420	chr4H	312,111,636	312,189,673	312,146,232	Simple RDA (p = 2.007e-06; q = 0.002)	-	ATP-dependent RNA helicase
				312,146,232	Partial RDA (p = 2.641e-04; q = 0.031)	-	
				312,146,215	LFMM (p = 1.940e-06; q = 0.028)	Latitude+Rain+Solar_rad	
				312,146,232	BAYPASS ($X^T X$ = 11.303)	-	
HORVU.MOREX.r2.4HG0314300	chr4H	392,019,895	392,026,683	392,022,112	Simple RDA (p = 3.183e-09; q = 3.245e-05)	-	Nucleolar GTP-binding protein 2
				392,022,112	Partial RDA (p = 5.620e-07; q = 0.001)	-	
				392,021,665	LFMM (p = 1.372e-4; q = 0.0364)	Elevation+Temperature	
				392,021,634	BAYPASS ($X^T X$ = 14.690)	-	

Table S8 Result of gene ontology (GO) term enrichment

	Enriched GO	P-value	FDR	GO definition
Simple RDA	GO:0004556	2.7640e-07	0.0014	Catalysis of the endohydrolysis of (1->4)-alpha-D-glucosidic linkages in polysaccharides containing three or more alpha-(1->4)-linked D-glucose units.
	GO:0103025	2.3737e-07	0.0014	Catalysis of the reaction: n H2O + a 1,4-alpha-D-glucan = alpha-maltohexaose + a 1,4-alpha-D-glucan
BAYPASS	GO:0006631	8.2636e-06	0.0077	The chemical reactions and pathways involving fatty acids, aliphatic monocarboxylic acids liberated from naturally occurring fats and oils by hydrolysis.
	GO:0009735	1.1465e-06	0.0029	Any process that results in a change in state or activity of a cell or an organism (in terms of movement, secretion, enzyme production, gene expression, etc.) as a result of a cytokinin stimulus.
	GO:0009236	2.5754e-05	0.0203	The chemical reactions and pathways resulting in the formation of cobalamin (vitamin B12), a water-soluble vitamin characterized by possession of a corrin nucleus containing a cobalt atom.
	GO:0090351	1.18776e-07	0.0004	The process whose specific outcome is the progression of the seedling over time, beginning with seed germination and ending when the first adult leaves emerge.
	GO:0006635	2.4051e-06	0.0038	A fatty acid oxidation process that results in the complete oxidation of a long-chain fatty acid. Fatty acid beta-oxidation begins with the addition of coenzyme A to a fatty acid, and occurs by successive cycles of reactions during each of which the fatty acid is shortened by a two-carbon fragment removed as acetyl coenzyme A; the cycle continues until only two or three carbons remain (as acetyl-CoA or propionyl-CoA respectively).
	GO:0009062	5.8859e-06	0.0063	The chemical reactions and pathways resulting in the breakdown of a fatty acid, any of the aliphatic monocarboxylic acids that can be liberated by hydrolysis from naturally occurring fats and oils. Fatty acids are predominantly straight-chain acids of 4 to 24 carbon atoms, which may be saturated or unsaturated; branched fatty acids and hydroxy fatty acids also occur, and very long chain acids of over 30 carbons are found in waxes.
	GO:0009845	1.1054e-07	0.0004	The physiological and developmental changes that occur in a seed commencing with water uptake (imbibition) and terminating with the elongation of the embryonic axis.
	GO:0004325	2.4976e-06	0.0038	Catalysis of the reaction: protoheme = Fe(2+) + protoporphyrin IX.
	GO:0046487	1.1351e-09	1.1649e-05	The chemical reactions and pathways involving glyoxylate, the anion of glyoxylic acid, HOC-COOH.
	GO:0019395	1.7175e-05	0.0147	The removal of one or more electrons from a fatty acid, with or without the concomitant removal of a proton or protons, by reaction with an electron-accepting substance, by addition of oxygen or by removal of hydrogen.

Supplementary Figures

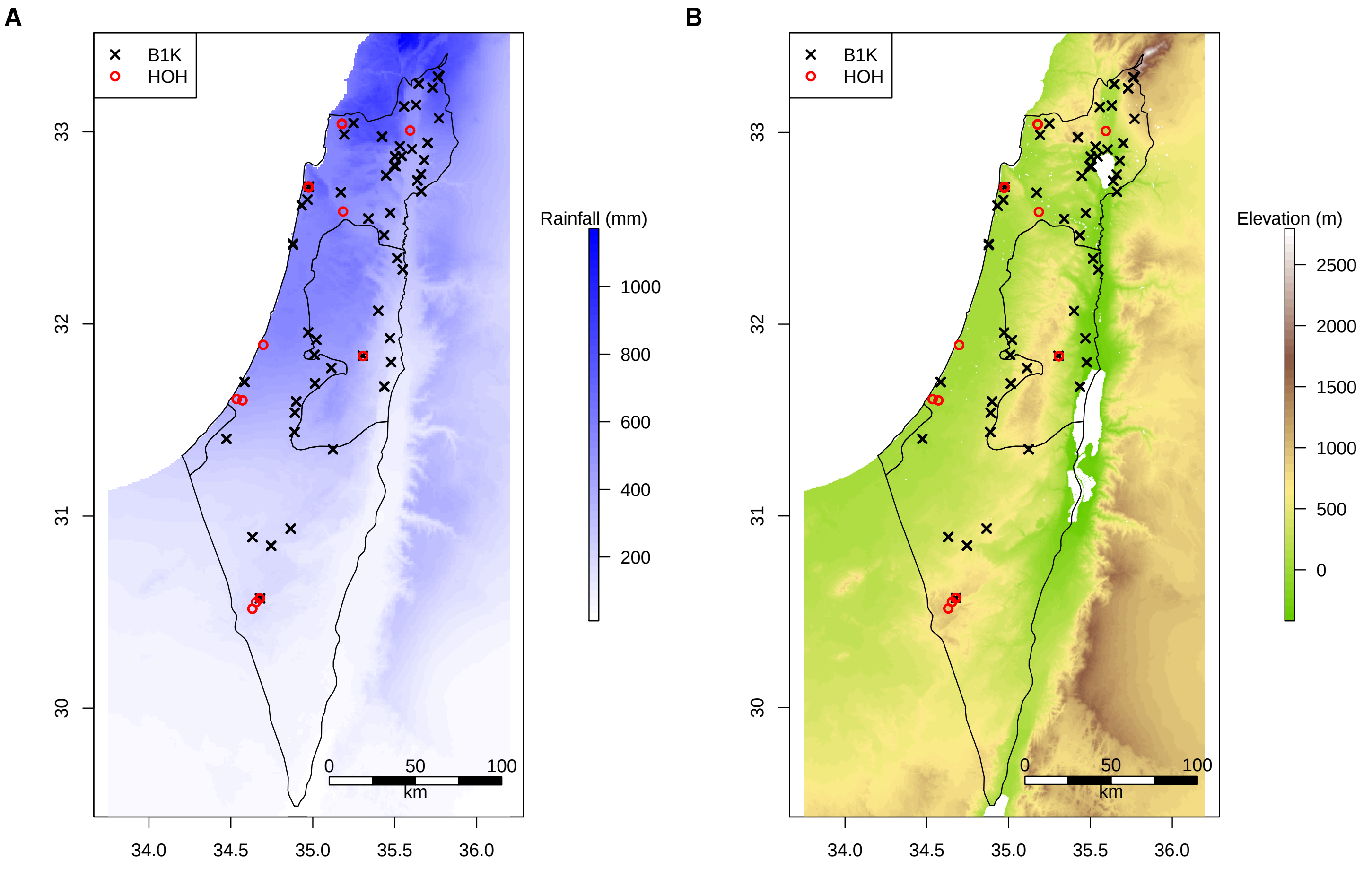

Figure S1 Collection sites of 244 B1K+ accessions. (A) Map with the accumulated rainfall between October and April. (B) Map with the geographical elevation. The black crosses and red circles represent the collection sites of B1K accessions and unpublished accessions stored at the University of Hohenheim (HOH collection), respectively

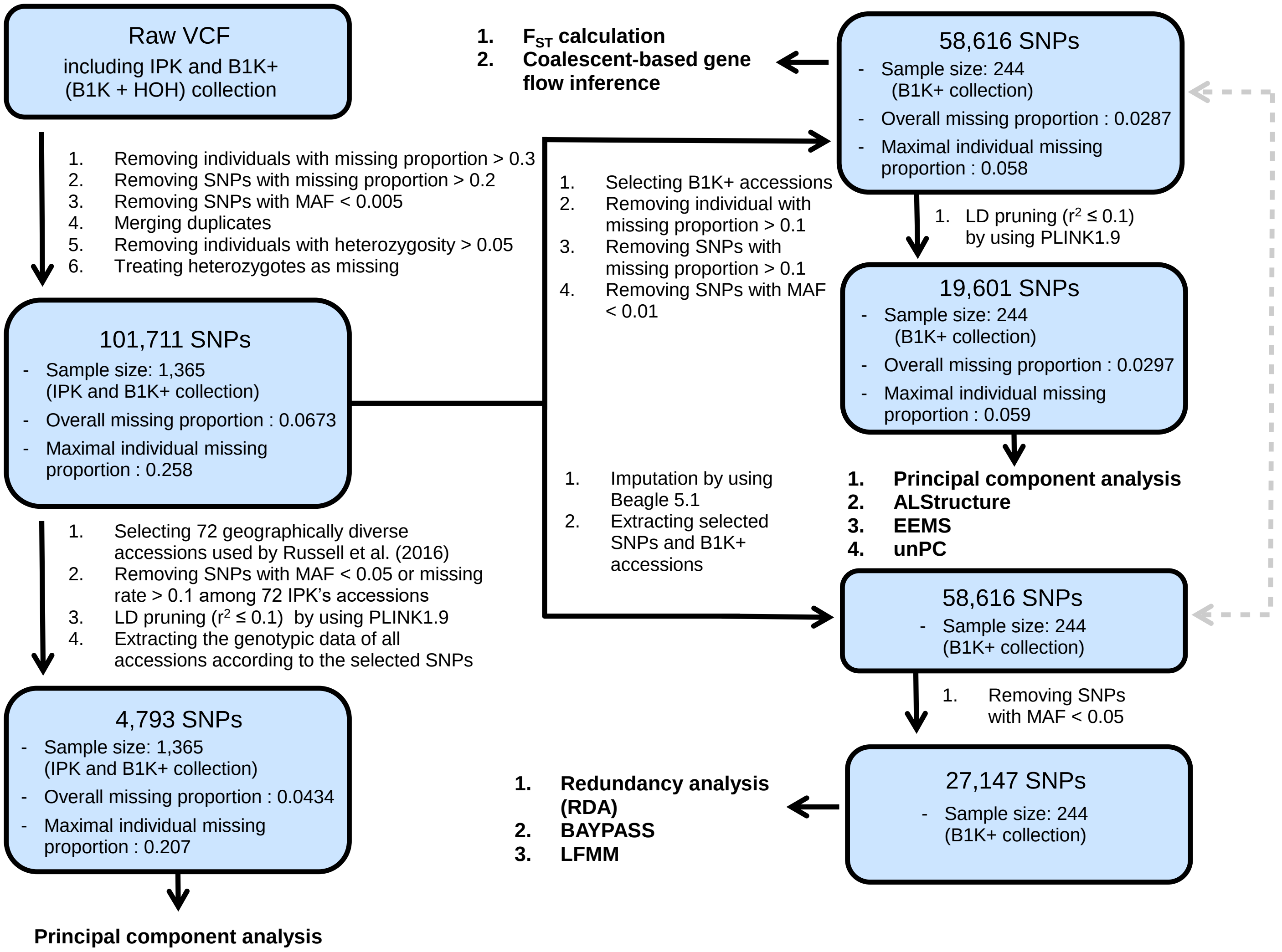

Figure S2 Workflow of genotypic data filtration. The double arrow with the gray dash line represents that two datasets are consisting of the same 58,616 SNP loci.

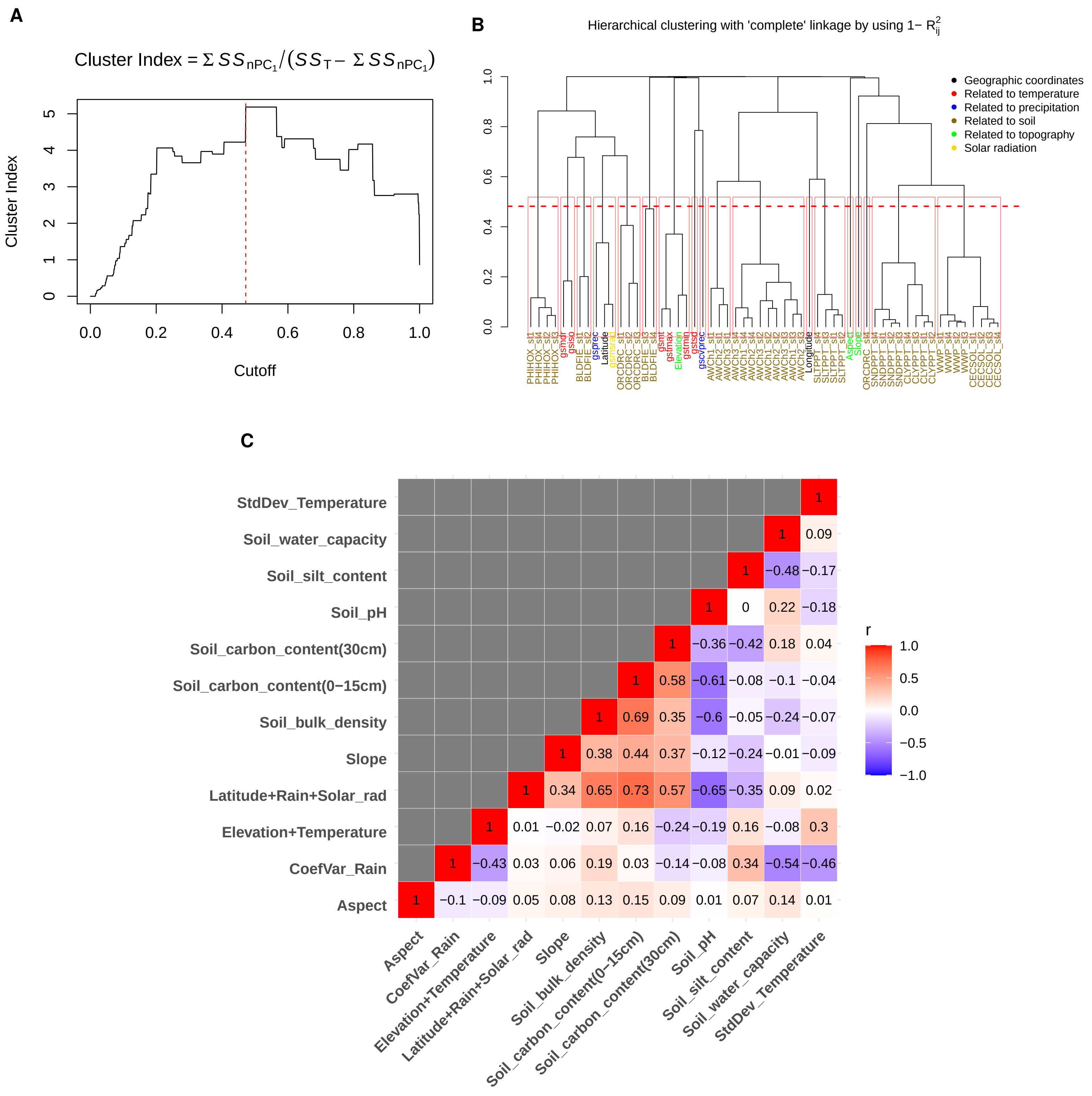
A
Cluster Index = $\Sigma SS_{nPC_1}/(SS_T - \Sigma SS_{nPC_1})$
Cluster Index
Cutoff
B
Hierarchical clustering with 'complete' linkage by using 1− R^2_{ij}
Geographic coordinates
Related to temperature
Related to precipitation
Related to soil
Related to topography
Solar radiation
C
StdDev_Temperature 1
Soil_water_capacity 1 0.09
Soil_silt_content 1 −0.48 −0.17
Soil_pH 1 0 0.22 −0.18
Soil_carbon_content(30cm) 1 −0.36 −0.42 0.18 0.04
Soil_carbon_content(0−15cm) 1 0.58 −0.61 −0.08 −0.1 −0.04
Soil_bulk_density 1 0.69 0.35 −0.6 −0.05 −0.24 −0.07
Slope 1 0.38 0.44 0.37 −0.12 −0.24 −0.01 −0.09
Latitude+Rain+Solar_rad 1 0.34 0.65 0.73 0.57 −0.65 −0.35 0.09 0.02
Elevation+Temperature 1 0.01 −0.02 0.07 0.16 −0.24 −0.19 0.16 −0.08 0.3
CoefVar_Rain 1 −0.43 0.03 0.06 0.19 0.03 −0.14 −0.08 0.34 −0.54 −0.46
Aspect 1 −0.1 −0.09 0.05 0.08 0.13 0.15 0.09 0.01 0.07 0.14 0.01
r
1.0
0.5
0.0
−0.5
−1.0

Figure S3

D

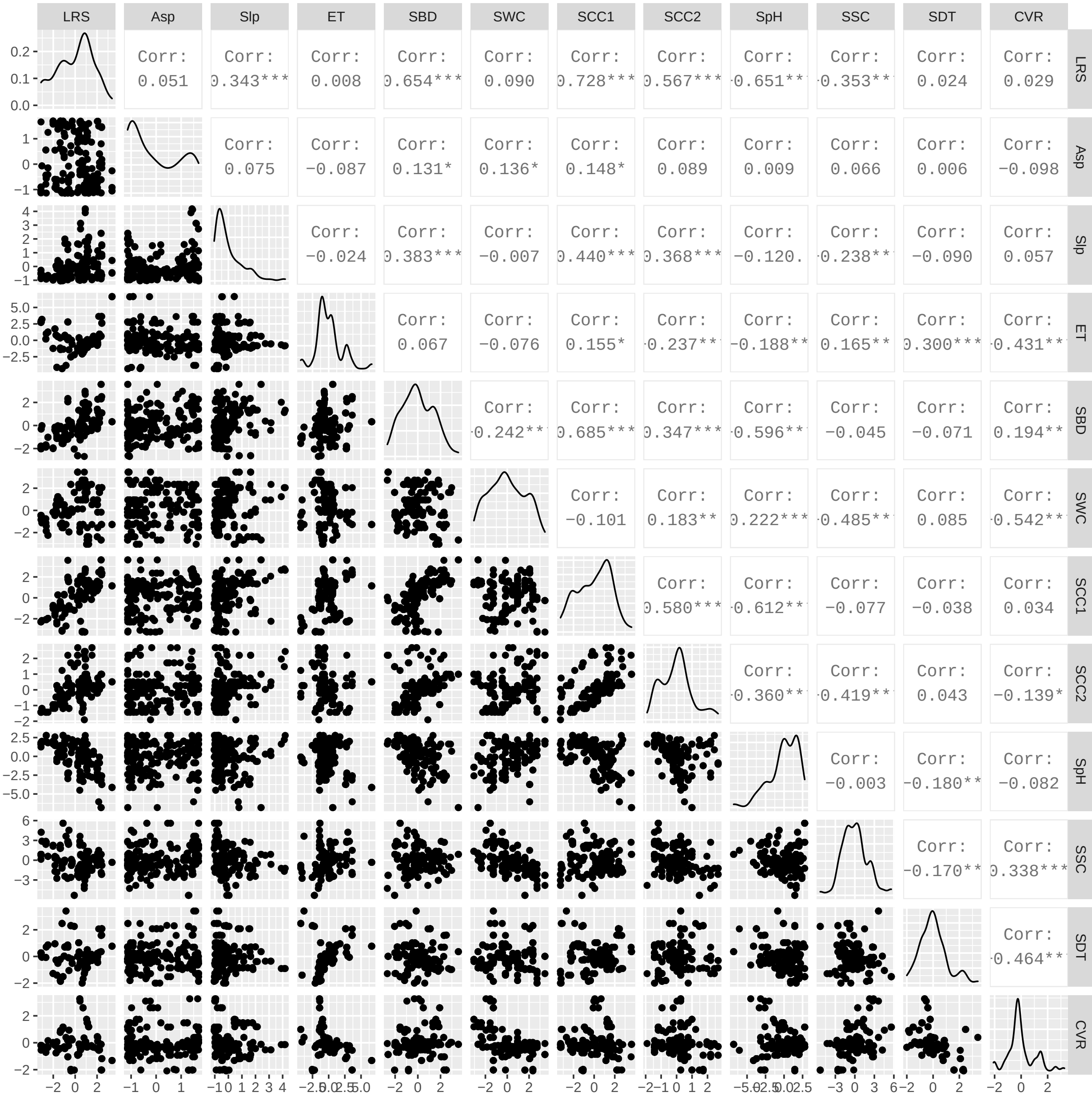

Figure S3 Procedure of generating synthetic environmental variables. (A) Relationship of cutoff for clustering and corresponding cluster index. The y-axis represents the customized cluster index, and the x-axis represents $1 - R^2$ that is used as a cutoff for the dendrogram. The vertical red dash line shows the optimal cutoff, which is 0.472 (B) Dendrogram of hierarchical clustering based on the $1 - R^2$. The horizontal red dash line shows the optimal cutoff used to determine the clustering combinations for generating synthetic environmental variables. (C) Correlation between the retained 12 environmental variables. (D) Scatterplot panel of the retained 12 environmental variables. Abbreviations: Asp:Aspect, CVR:CoefVar_Rain, ET:Elevation+Temperature, LRS:Latitude+Rain+Solar_rad, Slp:Slope, SBD:Soil_bulk_density, SCC1:Soil_carbon_content(0-15cm), SCC2:Soil_carbon_content(30cm), SpH:Soil_pH, SSC:Soil_silt_content, SWC:Soil_water_capacity, SDT:StdDev_Temperature

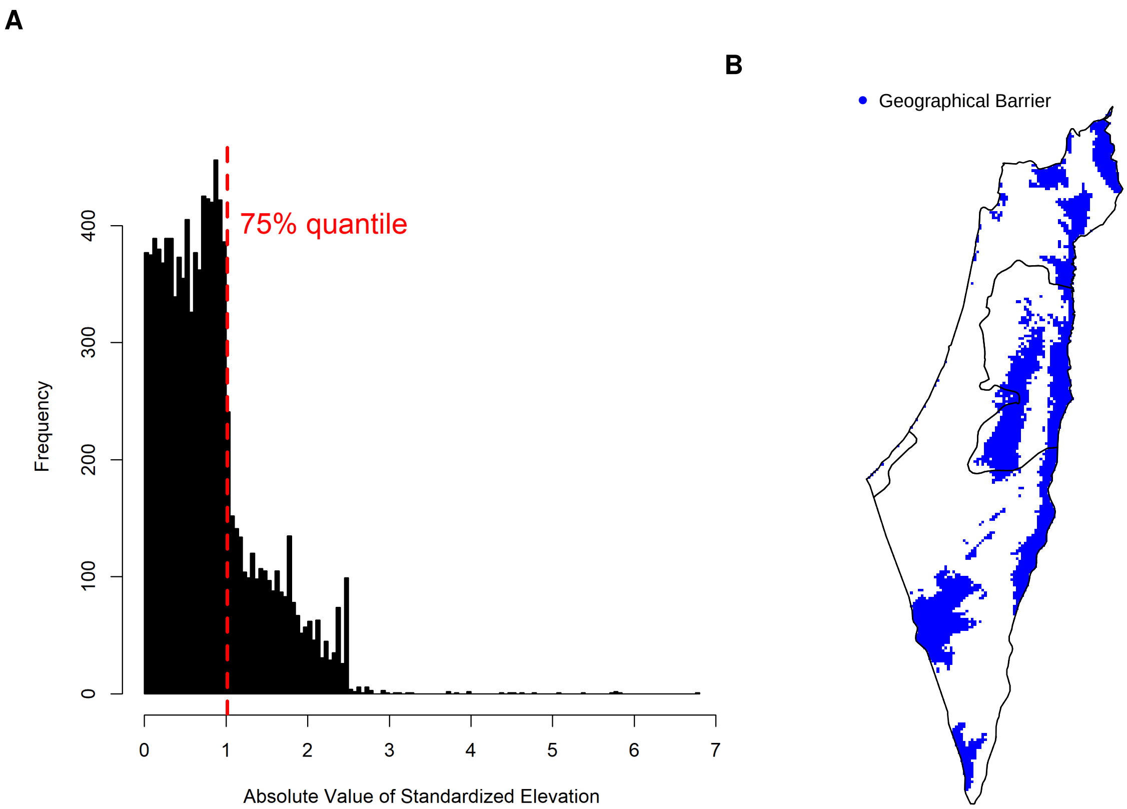

Figure S4 Classification of barrier and non-barrier pixels. (A) Histogram of absolute value of standardized elevation. The map pixels with elevation deviating from the majority of pixels are defined as geographical barriers. The top 25% cutoff is determined by the drastic decrease of pixel counts. (B) Geographical distribution of barrier pixels.

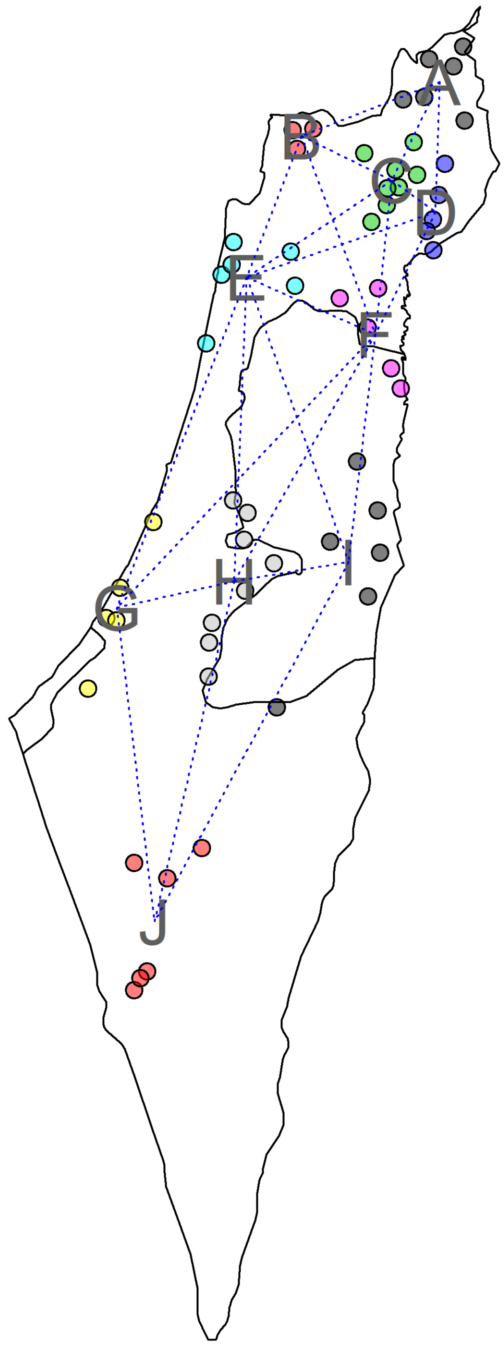

Figure S5 Geographical clusters and network used for the coalescent-based gene flow inference. The blue dash lines represent the edges of network which allows the movement between the connected geographical regions in the gene flow model.

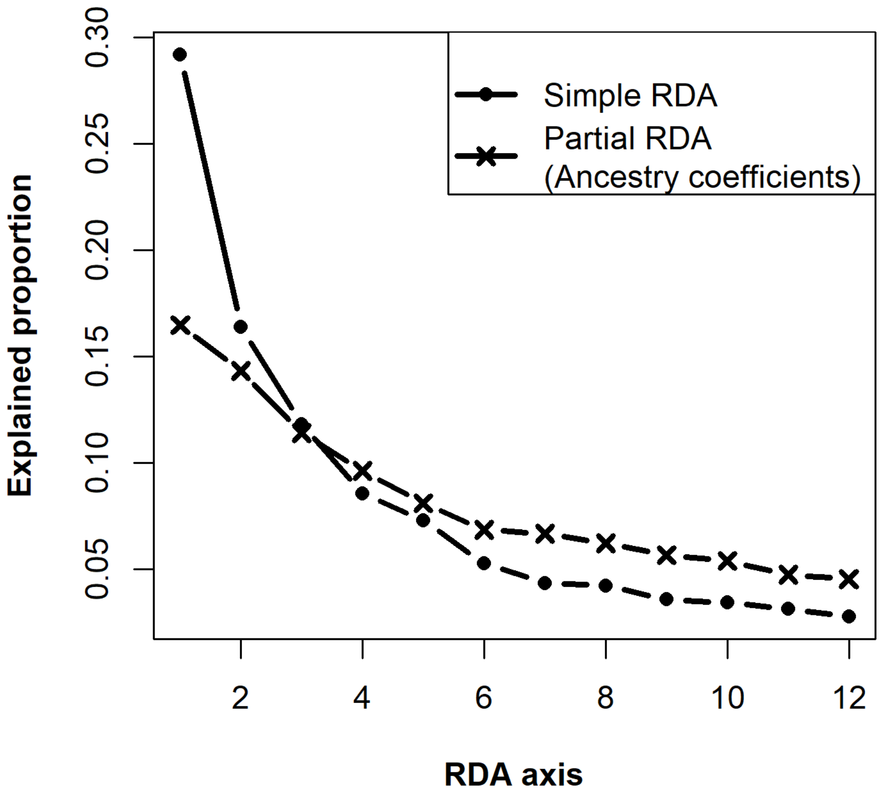

Figure S6 Explained proportion of variation of RDA axes. The lines with dots and crosses show the result of simple RDA and partial RDA conditioned on population structure, respectively. The first four RDA axes are selected to compute Mahalanobis distances.

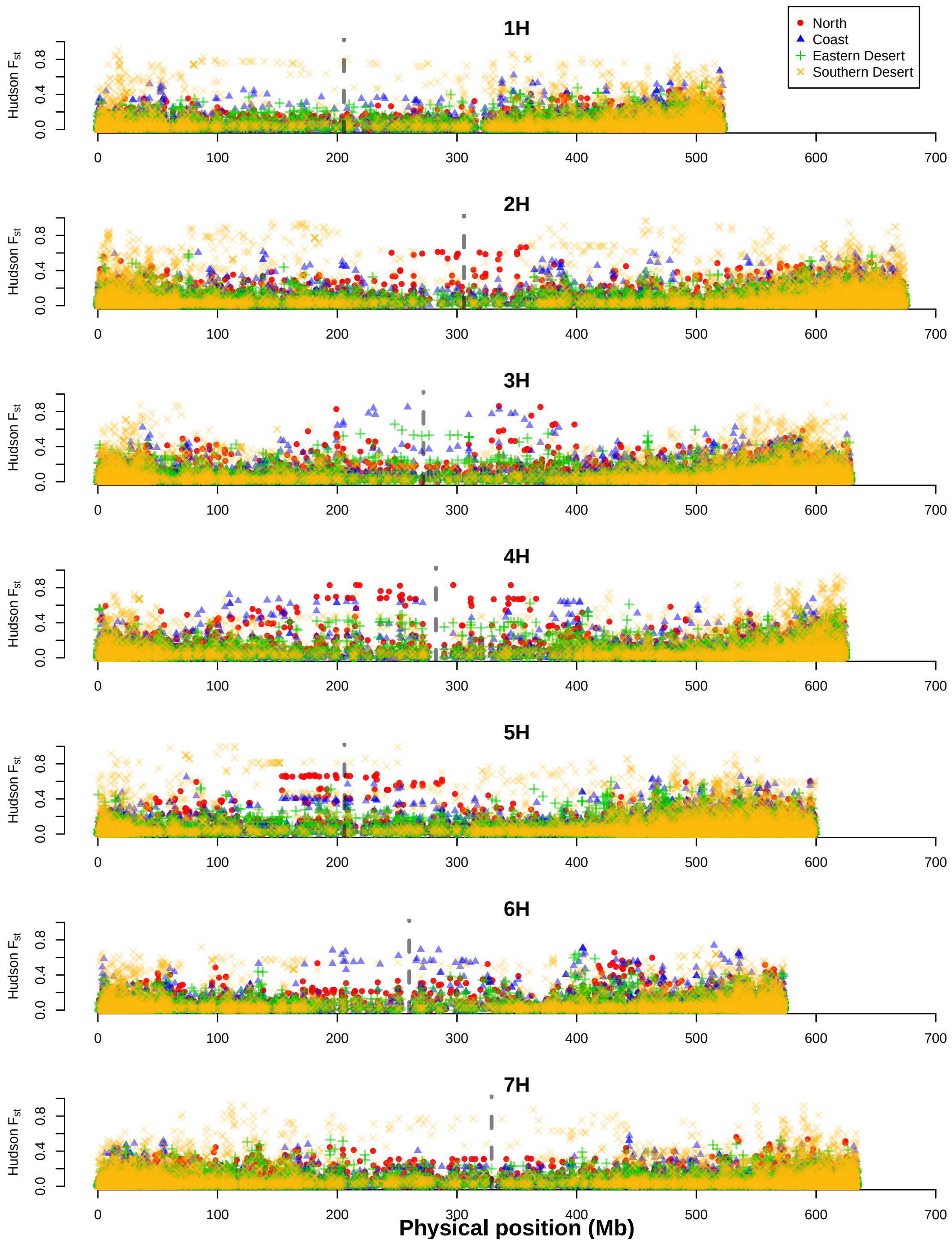

Figure S7 F_{ST} values along genome. The position of centromeres is shown by the vertical gray dash lines. The dots represent F_{ST} calculated between a specific genetic cluster and three remaining genetic clusters as a whole.

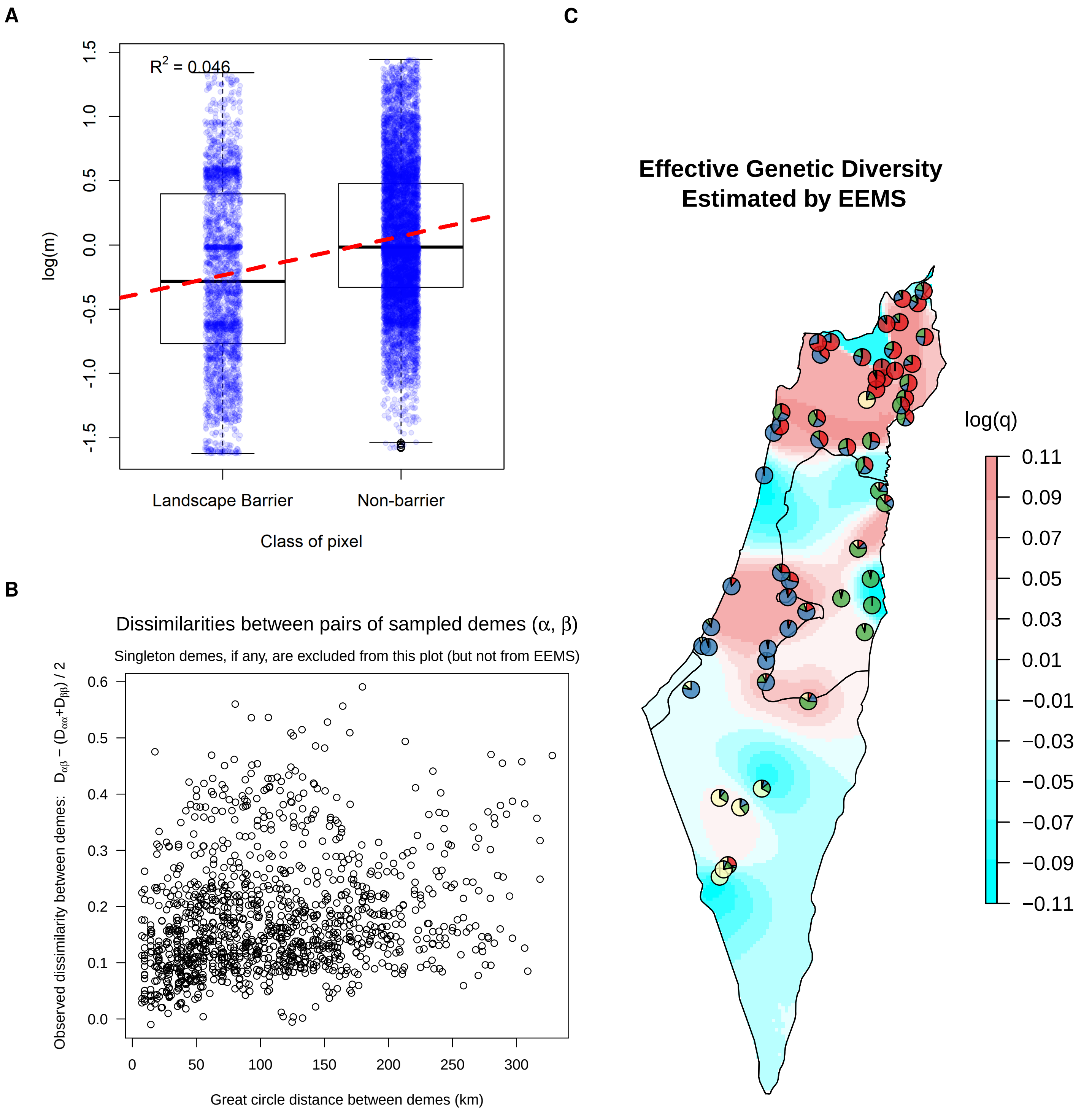

Figure S8 Result of EEMS. (A) Logarithm of gene flow rate ($log[m]$) estimated by EEMS classified according to barrier and non-barrier pixels. (B) Relationship between observed genetic dissimilarity computed by EEMS and geographical distances. (C) Effective genetic diversity surface estimated by EEMS. Effective genetic diversity (q) is the expected genetic dissimilarity of two individuals sampled from a location. The pie charts represent the average ancestry coefficients of accessions in each deme.

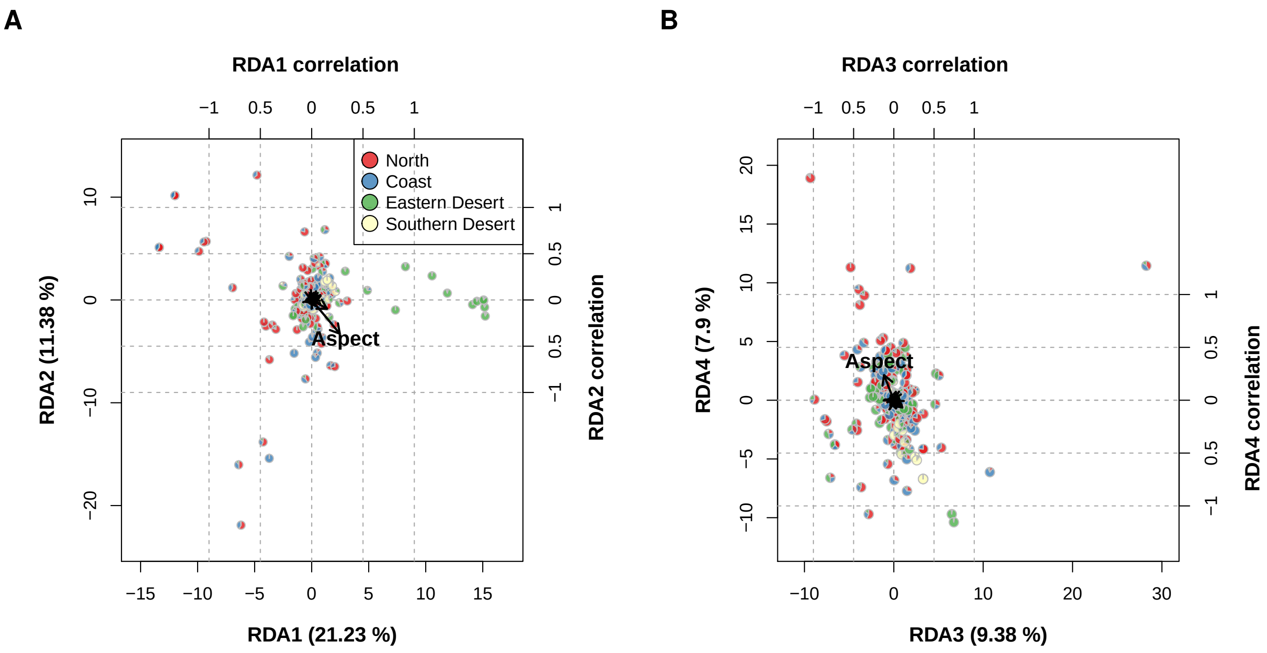

Figure S9 Biplot of RDA conditioned on spatial autocorrelation. (A) Biplot of the first and second RDA axes. (B) Biplot of the third and fourth RDA axes. The arrows represent correlations of the environmental variables with RDA axes that are shown in Table S6 in details. The pies represent ancestry coefficients of individuals. The coordinates of pies correspond to site scores of individuals on RDA axes.

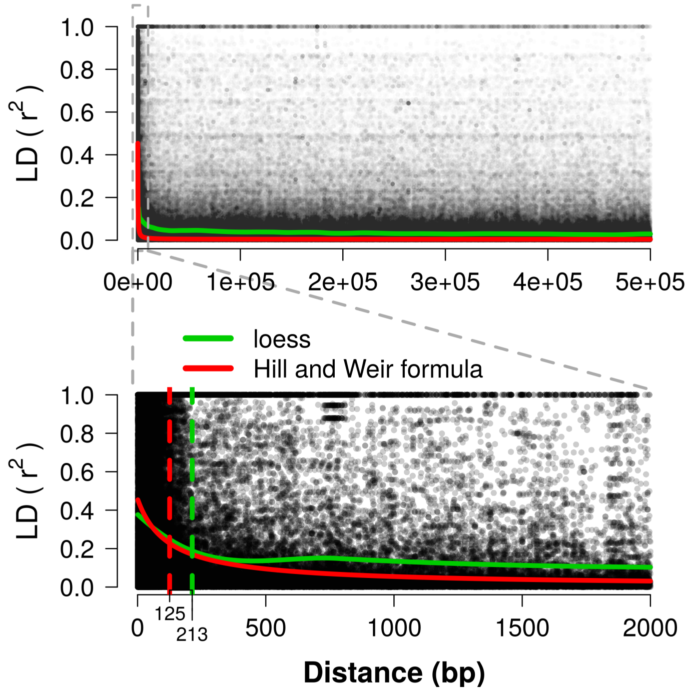

Figure S10 Linkage disequilibrium decay of the B1K+ collection across genome zooming in the first 500 kb and 2 kb. The green and red lines represent the fitted r^2 values by using *loess* method and Hill and Weir formula. The green and red dash lines represent the decay to the half of the highest fitted r^2.

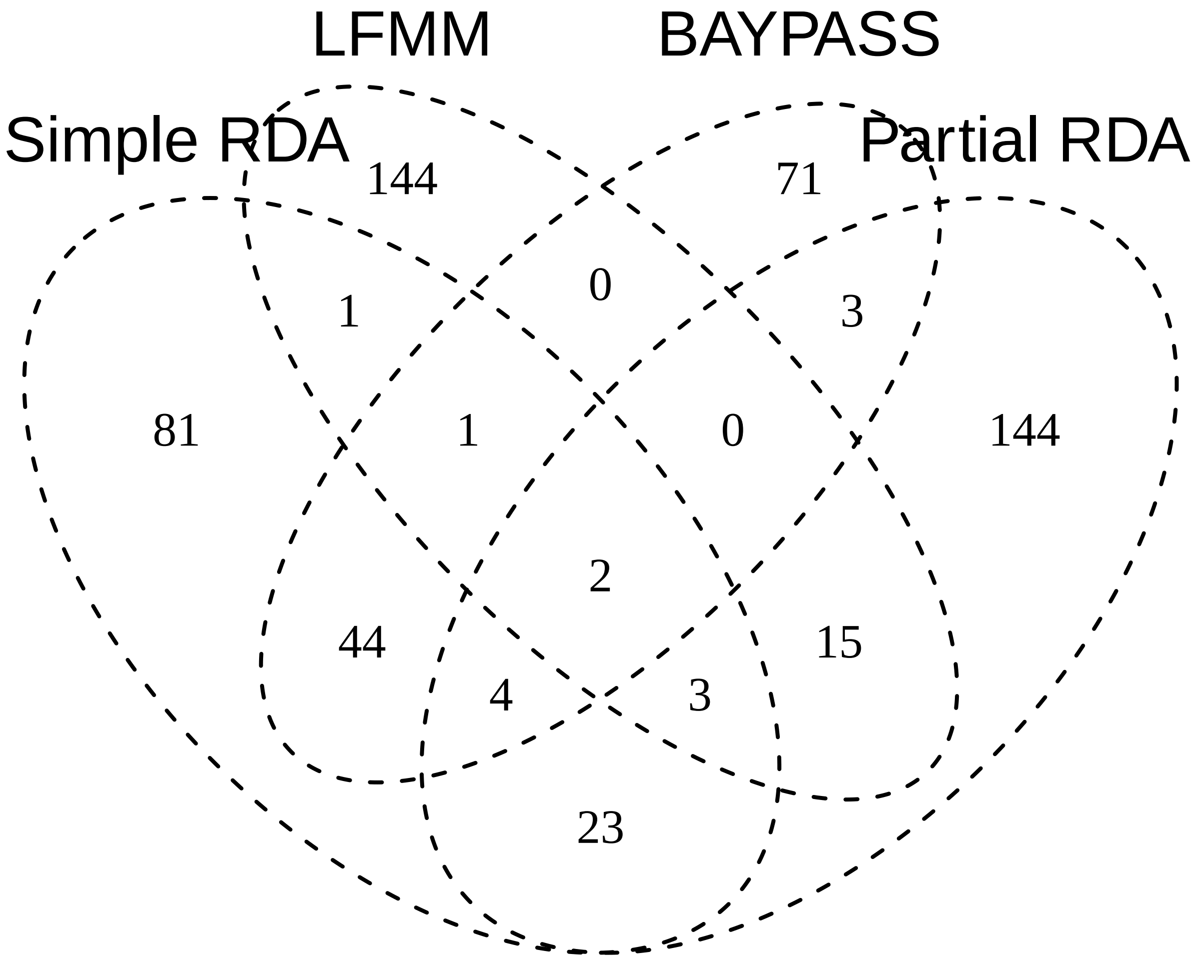

Figure S11 Number of genes locating within 500 bp adjacent intervals of significant SNPs detected by the GEA and outlier methods.

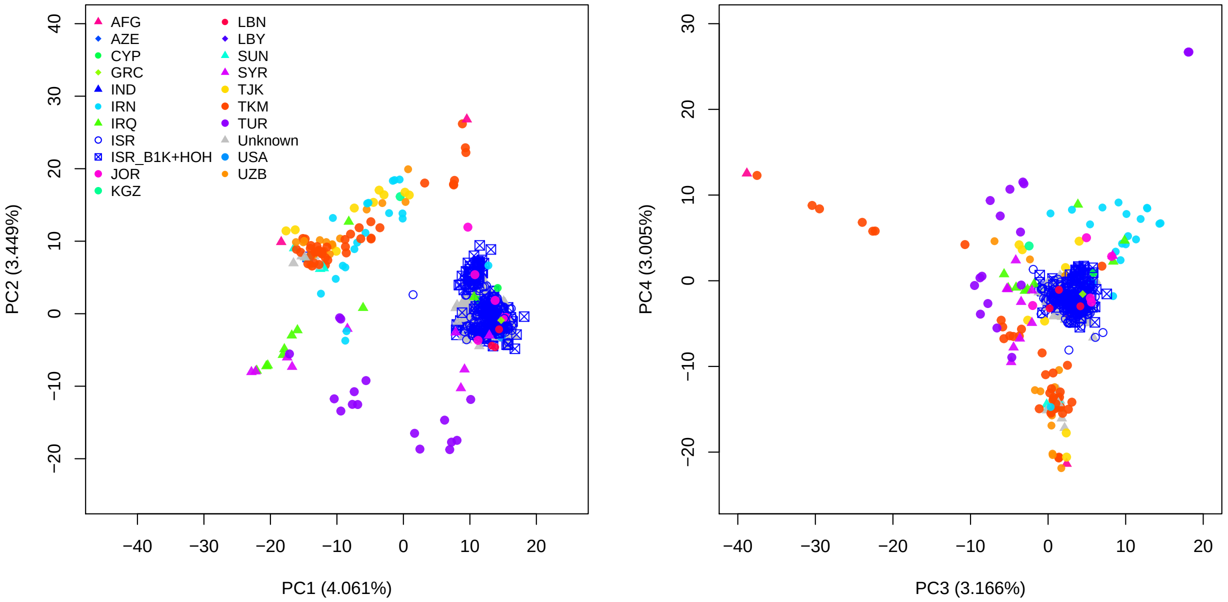

Figure S12 PCA plots of 244 B1K+ accessions and 1,121 accessions from IPK's genebank with countries of origins. (A) Result of PCA performed by using all of the available accessions. (B) Result of PCA performed by using 72 geographically diverse accessions and projecting the remaining accessions to PC spaces. The 244 B1K+ accessions are represented by blue boxes with crosses inside. The blue open dots represent IPK accessions originating in Israel and the gray closed triangles represent IPK accessions with unknown origins. The country abbreviations are according to ISO 3166-1 α-3.

Supplementary Files

File S1 Geographical coordinates and environmental data of 244 B1K+ used in this study. Both raw environmental data and twelve environmental variables used in genome-environment association analysis are included in the file. Grouping information of 58 demes and 10 manually defined regions used in gene flow analysis are also included.

File S2 A demonstration showing the influence of marker number and additional samples (HOH accessions) on the result of population structure analyses.

File S3 Statistics of the genome-wide scan methods for 27,147 SNPs, including $X^T X$ of *BAYPASS*, and the p and q values of the simple RDA, partial RDA and LFMM.

File S4 Annotations of genes in the upstream or downstream 500 bp of the candidate SNPs detected by the genome scan methods (*BAYPASS*, simple RDA, partial RDA and LFMM).

File S5 A table of European Nucleotide Archive (ENA) sample IDs.

References

Brown, A., Zohary, D., and Nevo, E. (1978). Outcrossing rates and heterozygosity in natural populations of hordeum spontaneum koch in israel. *Heredity*, 41(1):49–62.

Browning, B. L., Zhou, Y., and Browning, S. R. (2018). A one-penny imputed genome from next-generation reference panels. *The American Journal of Human Genetics*, 103(3):338–348.

Cabreros, I. and Storey, J. D. (2019). A likelihood-free estimator of population structure bridging admixture models and principal components analysis. *Genetics*, 212(4):1009–1029.

Fick, S. E. and Hijmans, R. J. (2017). Worldclim 2: new 1-km spatial resolution climate surfaces for global land areas. *International journal of climatology*, 37(12):4302–4315.

Hengl, T., Mendes de Jesus, J., Heuvelink, G. B., Ruiperez Gonzalez, M., Kilibarda, M., Blagotić, A., Shangguan, W., Wright, M. N., Geng, X., Bauer-Marschallinger, B., et al. (2017). Soilgrids250m: Global gridded soil information based on machine learning. *PLoS one*, 12(2):e0169748.

Hübner, S., Höffken, M., Oren, E., Haseneyer, G., Stein, N., Graner, A., Schmid, K., and Fridman, E. (2009). Strong correlation of wild barley (hordeum spontaneum) population structure with temperature and precipitation variation. *Molecular Ecology*, 18(7):1523–1536.

Legendre, P. and Legendre, L. (2012). Canonical analysis. In *Numerical ecology, 3rd English Edition*, chapter 11, page 558. The Netherlands: Elsevier Science BV.

Milner, S. G., Jost, M., Taketa, S., Mazón, E. R., Himmelbach, A., Oppermann, M., Weise, S., Knüpffer, H., Basterrechea, M., König, P., et al. (2019). Genebank genomics highlights the diversity of a global barley collection. *Nature genetics*, 51(2):319–326.

Purcell, S., Neale, B., Todd-Brown, K., Thomas, L., Ferreira, M. A., Bender, D., Maller, J., Sklar, P., De Bakker, P. I., Daly, M. J., et al. (2007). Plink: a tool set for whole-genome association and population-based linkage analyses. *The American journal of human genetics*, 81(3):559–575.

Russell, J., Mascher, M., Dawson, I. K., Kyriakidis, S., Calixto, C., Freund, F., Bayer, M., Milne, I., Marshall-Griffiths, T., Heinen, S., et al. (2016). Exome sequencing of geographically diverse barley landraces and wild relatives gives insights into environmental adaptation. *Nature Genetics*, 48(9):1024.
