# Supplementary material for: Physical geography, isolation by distance and environmental variables shape genomic variation of wild barley (Hordeum vulgare L. ssp. spontaneum) in the Southern Levant
Source: Heredity (Edinb). 2022 Jan 11;128(2):107–19. doi: 10.1038/s41437-021-00494-x (PMC8814169; doi:10.1038/s41437-021-00494-x)
Supplement: Supplementary file 3 — File S2 [file 41437_2021_494_MOESM3_ESM.html]

File S2


# File S2

#### Che-Wei Chang

#### August 9, 2021

## Load genotypic data

```
library(vcfR)

# a function to convert VCF to a numeric matrix
vcf_to_nummatrix <- function(vcf){
  require(vcfR)
  out <-
    apply(extract.gt(vcf), 2, function(x){
      c(0,0,1,1,1,2,2)[match(x, c("0/0", "0|0", "0/1", "0|1","1|0", "1/1", "1|1"))]
    })
  return(out)
} # vcf_to_nummatrix end


# load VCF
vcf <- read.vcfR("./2020-04-07-GBS_191B1K+53KS_19601SNP.vcf.gz", verbose = F)
Xmat <- vcf_to_nummatrix(vcf = vcf)
impX <- t(apply(Xmat,1,function(x){
  x[is.na(x)] <- mean(x, na.rm = T)
  return(x)
}))

## load ancestry coefficients estimated with the complete dataset
alsQ <- t(readRDS("./2020-04-09-ALStructure_result_191B1K+53KS.RDS")$Q_hat)
```

## PCA plot with the complete data

**Please note that the colors of points in the 3D plot are assigned according the highest acestry coefficients estimated with K=4 as described in the Figure 1 of the manuscript.**

```
# PCA with complete data
barley.pc <- prcomp(t(impX))


plot(x = barley.pc$x[,1], y = barley.pc$x[,2], pch = c(3,4)[grepl(colnames(impX), pattern = "Kjs|KARL|IL") + 1],
     col = c("red", "blue")[grepl(colnames(impX), pattern = "Kjs|KARL|IL") + 1],
     xlab = "PC1", ylab = "PC2", main = "Complete data (19,601 SNPs and 244 accessions)")
legend("bottomleft", col = c("red", "blue"), pch = c(3,4), legend = c("B1K", "HOH"))
```

```
# make 3D plot
library(ggplot2)
library(scales)
mycol <- alpha(c('#e41a1c','#377eb8','#4daf4a', '#ffffbf'), alpha = 0.8)
g.type <- apply(alsQ, 1, function(x){colnames(alsQ)[which.max(x)]}) # assign the type of genetic cluster according to the highest Q value
df <- data.frame(type = g.type,barley.pc$x[,1:3])
```

```
PC1 <- df$PC1
PC2 <- df$PC2
PC3 <- df$PC3
par3d(windowRect = c(100, 100, 600, 350))
plot3d(PC1, PC2, PC3, bg = mycol[match(df$type, c("North", "Coast","Eastern Desert", "Southern Desert"))], pch =NULL , col = "white")
pch3d(PC1, PC2, PC3, bg = mycol[match(df$type, c("North", "Coast","Eastern Desert", "Southern Desert"))], pch =21 , col = 1, cex = 0.5)
legend3d("topright", legend = c("North", "Coast","Eastern Desert", "Southern Desert"), col = mycol, pch = 16)
```

## PCA with subset SNPs

### Randomly select 100 SNPs

```
# PCA with a dataset containing only 100 SNPs
set.seed(1234)
marker100 <- sample(1:nrow(impX), size = 100, replace = F) # randomly select 100 SNPs
barley.pc.subM100 <- prcomp(t(impX[marker100,]))

plot(x = barley.pc.subM100$x[,1], y = barley.pc.subM100$x[,2], pch = c(3,4)[grepl(colnames(impX), pattern = "Kjs|KARL|IL") + 1],
     col = c("red", "blue")[grepl(colnames(impX), pattern = "Kjs|KARL|IL") + 1],
     xlab = "PC1", ylab = "PC2", main = "With subset SNPs (100 SNPs and 244 accessions)")
```

```
df <- data.frame(type = g.type,barley.pc.subM100$x[,1:3])
```

```
PC1 <- df$PC1
PC2 <- df$PC2
PC3 <- df$PC3
plot3d(PC1, PC2, PC3, bg = mycol[match(df$type, c("North", "Coast","Eastern Desert", "Southern Desert"))], pch =NULL , col = "white")
pch3d(PC1, PC2, PC3, bg = mycol[match(df$type, c("North", "Coast","Eastern Desert", "Southern Desert"))], pch =21 , col = 1, cex = 0.5)
legend3d("topright", legend = c("North", "Coast","Eastern Desert", "Southern Desert"), col = mycol, pch = 16)
```

### Randomly select 5,000 SNPs

```
set.seed(1234)
marker5000 <- sample(1:nrow(impX), size = 5000, replace = F) # randomly select 5000 SNPs
barley.pc.subM5000 <- prcomp(t(impX[marker5000,]))

df <- data.frame(type = g.type,barley.pc.subM5000$x[,1:3])

plot(x = barley.pc.subM5000$x[,1], y = barley.pc.subM5000$x[,2], pch = c(3,4)[grepl(colnames(impX), pattern = "Kjs|KARL|IL") + 1],
     col = c("red", "blue")[grepl(colnames(impX), pattern = "Kjs|KARL|IL") + 1],
     xlab = "PC1", ylab = "PC2", main = "With subset SNPs (5000 SNPs and 244 accessions)")
```

```
PC1 <- df$PC1
PC2 <- df$PC2
PC3 <- df$PC3
plot3d(PC1, PC2, PC3, bg = mycol[match(df$type, c("North", "Coast","Eastern Desert", "Southern Desert"))], pch =NULL , col = "white")
pch3d(PC1, PC2, PC3, bg = mycol[match(df$type, c("North", "Coast","Eastern Desert", "Southern Desert"))], pch =21 , col = 1, cex = 0.5)
legend3d("topright", legend = c("North", "Coast","Eastern Desert", "Southern Desert"), col = mycol, pch = 16)
```

## PCA without HOH accessions (using only 191 B1Ks with 19,601 SNPs)

```
# PCA with a dataset exluding HOH accessions
barley.pc.noHOH <- prcomp(t(impX[,-grep(colnames(impX), pattern = "Kjs|KARL|IL")]))

plot(x = barley.pc.noHOH$x[,1], y = barley.pc.noHOH$x[,2], pch = 3,
     col = "red",
     xlab = "PC1", ylab = "PC2", main = "Without HOH (19,601 SNPs and 191 B1K accessions)")
```

```
alsQ.sub <- alsQ[-grep(colnames(impX), pattern = "Kjs|KARL|IL"),]
g.type <- apply(alsQ.sub, 1, function(x){colnames(alsQ.sub)[which.max(x)]}) # assign the type of genetic cluster according to the highest Q value
df <- data.frame(type = g.type,barley.pc.noHOH$x[,1:6])
```

#### PC1 + PC2 + PC3

```
PC1 <- df$PC1
PC2 <- df$PC2
PC3 <- df$PC3
plot3d(PC1, PC2, PC3, bg = mycol[match(df$type, c("North", "Coast","Eastern Desert", "Southern Desert"))], pch =NULL , col = "white")
pch3d(PC1, PC2, PC3, bg = mycol[match(df$type, c("North", "Coast","Eastern Desert", "Southern Desert"))], pch =21 , col = 1, cex = 0.5)
legend3d("topright", legend = c("North", "Coast","Eastern Desert", "Southern Desert"), col = mycol, pch = 16)
```

#### PC1 + PC4 + PC5

The eastern and southern desert clusters separate in the PC4 even though HOH accessions are excluded.

```
PC1 <- df$PC1
PC4 <- df$PC4
PC5 <- df$PC5
plot3d(PC1, PC4, PC5, bg = mycol[match(df$type, c("North", "Coast","Eastern Desert", "Southern Desert"))], pch =NULL , col = "white")
pch3d(PC1, PC4, PC5, bg = mycol[match(df$type, c("North", "Coast","Eastern Desert", "Southern Desert"))], pch =21 , col = 1, cex = 0.5)
legend3d("topright", legend = c("North", "Coast","Eastern Desert", "Southern Desert"), col = mycol, pch = 16)
```

## PCA without HOH accessions and with subset SNPs

### Randomly select 100 SNPs

```
# PCA with a dataset containing only 100 SNPs
set.seed(1234)
marker100 <- sample(1:nrow(impX), size = 100, replace = F) # randomly select 100 SNPs
barley.pc.noHOH.subM100 <- prcomp(t(impX[marker100, -grep(colnames(impX), pattern = "Kjs|KARL|IL")]))

plot(x = barley.pc.noHOH.subM100$x[,1], y = barley.pc.noHOH.subM100$x[,2], pch = 3,
     col = c("red"),
     xlab = "PC1", ylab = "PC2", main = "With subset SNPs (100 SNPs and 191 B1K accessions)")
```

```
df <- data.frame(type = g.type,barley.pc.noHOH.subM100$x[,1:3])
```

```
PC1 <- df$PC1
PC2 <- df$PC2
PC3 <- df$PC3
plot3d(PC1, PC2, PC3, bg = mycol[match(df$type, c("North", "Coast","Eastern Desert", "Southern Desert"))], pch =NULL , col = "white")
pch3d(PC1, PC2, PC3, bg = mycol[match(df$type, c("North", "Coast","Eastern Desert", "Southern Desert"))], pch =21 , col = 1, cex = 0.5)
legend3d("topright", legend = c("North", "Coast","Eastern Desert", "Southern Desert"), col = mycol, pch = 16)
```

### Randomly select 5,000 SNPs

```
set.seed(1234)
marker5000 <- sample(1:nrow(impX), size = 5000, replace = F) # randomly select 5000 SNPs
barley.pc.noHOH.subM5000 <- prcomp(t(impX[marker5000, -grep(colnames(impX), pattern = "Kjs|KARL|IL")]))

df <- data.frame(type = g.type,barley.pc.noHOH.subM5000$x[,1:5])

plot(x =barley.pc.noHOH.subM5000$x[,1], y = barley.pc.noHOH.subM5000$x[,2], pch = 3,
     col = c("red"),
     xlab = "PC1", ylab = "PC2", main = "With subset SNPs (5000 SNPs and 244 accessions)")
```

#### PC1 + PC2 + PC3

```
PC1 <- df$PC1
PC2 <- df$PC2
PC3 <- df$PC3
plot3d(PC1, PC2, PC3, bg = mycol[match(df$type, c("North", "Coast","Eastern Desert", "Southern Desert"))], pch =NULL , col = "white")
pch3d(PC1, PC2, PC3, bg = mycol[match(df$type, c("North", "Coast","Eastern Desert", "Southern Desert"))], pch =21 , col = 1, cex = 0.5)
legend3d("topright", legend = c("North", "Coast","Eastern Desert", "Southern Desert"), col = mycol, pch = 16)
```

#### PC1 + PC4 + PC5

```
PC1 <- df$PC1
PC4 <- df$PC4
PC5 <- df$PC5
plot3d(PC1, PC4, PC5, bg = mycol[match(df$type, c("North", "Coast","Eastern Desert", "Southern Desert"))], pch =NULL , col = "white")
pch3d(PC1, PC4, PC5, bg = mycol[match(df$type, c("North", "Coast","Eastern Desert", "Southern Desert"))], pch =21 , col = 1, cex = 0.5)
legend3d("topright", legend = c("North", "Coast","Eastern Desert", "Southern Desert"), col = mycol, pch = 16)
```

## Identify optimal K with `ALStructure`

`ALStructure` is used to estimate the optimal number of K for the six datasets above.

```
library(alstructure)
## with HOH accessions
d <- alstructure::estimate_d(impX)
d_subM100 <- alstructure::estimate_d(impX[marker100,])
```

```
## Warning in alstructure::estimate_d(impX[marker100, ]): d = 1 estimated: a
## minimum of d = 2 required
```

```
d_subM5000 <- alstructure::estimate_d(impX[marker5000,])
## without HOH accessions
d_noHOH <- alstructure::estimate_d(impX[,-grep(colnames(impX), pattern = "Kjs|KARL|IL")])
d_subM100.noHOH <- alstructure::estimate_d(impX[marker100,-grep(colnames(impX), pattern = "Kjs|KARL|IL")])
```

```
## Warning in alstructure::estimate_d(impX[marker100, -grep(colnames(impX), : d = 1
## estimated: a minimum of d = 2 required
```

```
d_subM5000.noHOH <- alstructure::estimate_d(impX[marker5000, -grep(colnames(impX), pattern = "Kjs|KARL|IL")])

d.result <- data.frame("SNP_number" = c(19601, 100, 5000, 19601, 100, 5000), 
                       "include_HOH" = c("Yes", "Yes", "Yes", "No", "No", "No"),
                       "K" = c(d, d_subM100, d_subM5000, d_noHOH, d_subM100.noHOH, d_subM5000.noHOH))

knitr::kable(d.result, align=rep('c', 3))
```

| SNP\_number | include\_HOH | K |
| --- | --- | --- |
| 19601 | Yes | 4 |
| 100 | Yes | 2 |
| 5000 | Yes | 4 |
| 19601 | No | 3 |
| 100 | No | 2 |
| 5000 | No | 3 |

The `ALStructure` suggested K=3 with 19,601 and 5,000 SNPs if the HOH accessions are exluded.
